# The resilience of *Salmonella* to bile stress is impaired due to the reduced efflux pump activity mediated by the antioxidant enzyme YqhD

**DOI:** 10.1128/msphere.00382-25

**Published:** 2025-10-02

**Authors:** Kirti Parmar, Yogyta Kumari, Raju S. Rajmani, Dipshikha Chakravortty

**Affiliations:** 1 Department of Microbiology and Cell Biology, Division of Biological Sciences, Indian Institute of Science, Bangalore, Karnataka, India; 2 Centre of Infectious Disease Research, Indian Institute of Science, Bangalore, Karnataka, India; 3 Adjunct Faculty, School of Biology, Indian Institute of Science Education and Research, Thiruvananthapuram, Kerala, India; University of Michigan Medical School, Ann Arbor, Michigan, USA

**Keywords:** *Salmonella* pathogenesis, bile stress, YqhD gene, redox

## Abstract

Bile salts play a critical role in modulating the host gut. They possess antimicrobial properties wherein they disrupt the bacterial membrane and produce reactive oxygen species (ROS), causing DNA damage. Pathogens like *Salmonella* regulate their metabolic activity to counteract the effects of bile. This study investigates the role of YqhD, an aldehyde reductase, in *Salmonella’*s bile salt susceptibility. Our findings reveal increased survival of the *yqhD* mutant in the *in-vitro* studies in LB media with bile, liver cell line HepG2 and C57BL/6 mice on treatment with 8% sodium cholate in the cecum. Bile salts, physiologically produced for the digestion of fat, enhanced the organ burden of the *yqhD* mutant in C57BL/6 mice on replacing the chow diet with a high-fat diet (HFD). The *yqhD* mutation, on bile salt exposure, also leads to increased ROS levels and modulation of antioxidant genes in the bacteria. The addition of the antioxidant glutathione during bile stress enhances the survival of STM WT and makes it similar to the survival of STM Δ*yqhD*. Similarly, in the *gp91^−/−phox^* mice, the organ burden and pathology of the liver and spleen were increased on STM WT infection, while it remained similar for the *yqhD* mutant on exposure to HFD compared to that of chow-fed mice. Furthermore, the *yqhD* mutant exhibited increased AcrAB efflux pump activity, regulated by RamA/R regulon.

**IMPORTANCE:**

Foodborne pathogen *Salmonella* can tolerate high concentrations of bile and even survive the harsh environment of the gall bladder. This study is significant as it explores the role of a novel antioxidant gene *yqhD* in bile salt susceptibility of *Salmonella* Typhimurium and Typhi. It highlights how the presence of gene *yqhD*, though advantageous in macrophages, reduces the *Salmonella* survival on bile salt exposure *in vitro* and in liver cell line HepG2. Deletion of *yqhD* increased the survival on bile stress exposure, which was attributed to its ability to induce the AcrAB efflux pump of *Salmonella*. A deeper understanding of how *Salmonella* modulates gene expression in response to bile stress could provide valuable insights into addressing the chronic carriage of *Salmonella*.

## INTRODUCTION

In modern times, the development of the fast-food industry has changed human lifestyle, and the high-fat diet (HFD) has become popular. The high-fat diet contains 36–40% fat (39). An HFD induces inflammation and dysbiosis of the gut health by increasing the ratio of *Firmicutes* to *Bacteroidetes*. It also increases the relative abundance of Enterobacteriaceae (1, 2). *Salmonella* is a Gram-negative, rod-shaped bacterium belonging to the *Enterobacteriaceae* family that infects various hosts (3). *Salmonella* is also a bile-resistant pathogen, and bile salt production by HFD provides an ambient condition for its growth (4). *Salmonella enterica* colonizes and persists in the harsh environment of the gallbladder during chronic infection (5). Bile is a digestive secretion synthesized by hepatocytes in the liver from cholesterol and stored in the gallbladder or directly secreted into the intestine. Physiologically, bile aids in the digestion of fats and the absorption of fat-soluble vitamins (6). Although antibacterial, they are used as environmental signals by bacteria to regulate their pathogenesis and bile resistance (7–11). In earlier studies, the role of major antioxidant genes in bile salt treatment was reported to be negligible except for disulfide stress, and their deletion did not confer any disadvantage to the bacteria (12).

In our study, we investigated the role of YqhD in *Salmonella enterica* serovars Typhimurium and Typhi. YqhD is an NADPH-dependent aldehyde reductase with zinc as a cofactor (13). Studies have shown that YqhD decreases reactive oxygen species (ROS) levels and ROS-mediated effects by acting on aldehydes such as propanal and butanal, which are generated upon membrane peroxidation and glyoxal that is produced by glucose oxidation (14, 15). A transcriptomics analysis study of *Salmonella* under varying stresses showed that *yqhD* was induced by iron deficiency, bile, and osmotic stress (16). For the first time, we decipher the role of YqhD in potentiating bile susceptibility in *Salmonella*. We show that *yqhD* deletion leads to increased survival in the presence of bile. However, bile increases ROS levels and the AcrAB efflux pump activity in STM Δ*yqhD*. Inhibition of the AcrAB efflux pump activity reverses the growth advantage of the *yqhD* mutant in bile stress. We delineate a novel mechanism determining sensitivity to bile in *Salmonella,* involving YqhD as a critical player by modulating the efflux pump activity through RamA.

## RESULTS

### *yqhD* gene reduced *Salmonella* survival on exposure to bile salts

**Fig 1:**
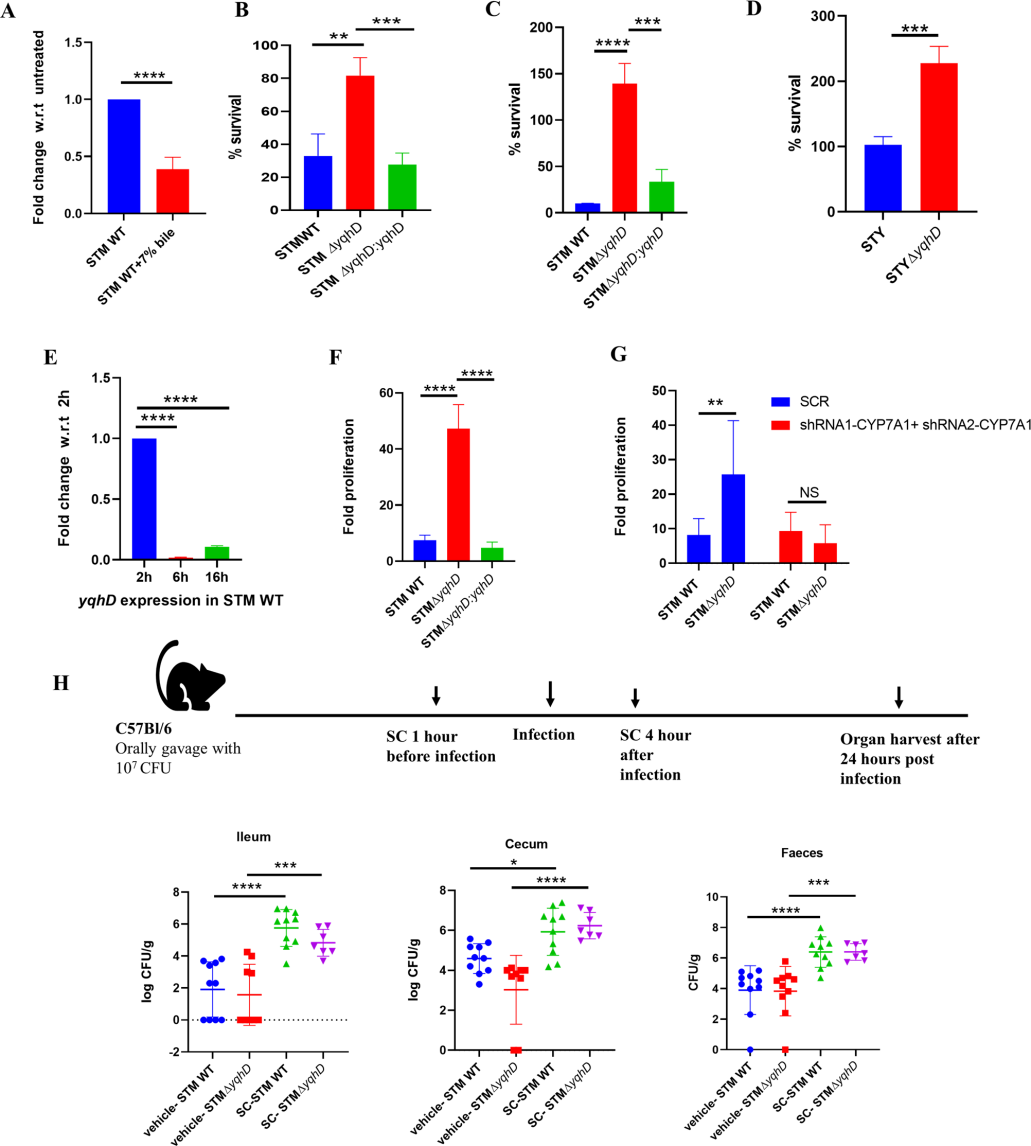
Deletion of *yqhD* in *Salmonella* increases growth on exposure to bile salts. (A) The mRNA expression of *yqhD* in STM WT on treatment with 7% bile with respect to untreated (*N* = 3, *n* = 3). Data are one representative result with mean ± SD, analysis by unpaired *t*-test. (B) The *in vitro* sensitivity assay on the treatment of 7% primary bile salt to STM WT, STMΔ*yqhD,* and STMΔ*yqhD:yqhD* normalized to un-treated by CFU/mL (*N* = 5, *n* = 2). Data are one representative result with mean ± SD, analysis by unpaired *t*-test. (C) The *in vitro* sensitivity assay on the treatment of 1% sodium deoxycholate (Sigma) to STM WT, STMΔ*yqhD,* and STMΔ*yqhD:yqhD* by CFU/mL (*N* = 3, *n* = 2). Data are one representative result with mean ± SD, analysis by unpaired *t*-test. (D) The *in vitro* sensitivity assay on the treatment of 7% primary bile salt to *Salmonella* Typhi CT18, *Salmonella* Typhi Δ*yqhD* by CFU/mL (*N* = 3, *n* = 2). Data are one representative result with mean ± SD, analysis by unpaired *t*-test. (E) The mRNA expression of *yqhD* in STM WT upon infection in the HepG2 cells with respect to STM WT (*N* = 3, *n* = 3). Data are one representative result with mean ± SD, analysis by one-way ANOVA. (F) Fold proliferation of STM WT, STMΔ*yqhD,* and STMΔ*yqhD:yqhD* in HepG2 cells (*N* = 5, *n* = 3). Data are one representative result with mean ± SD, analysis by unpaired Student’s *t*-test. (G) Fold proliferation STM WT, STMΔ*yqhD* in HepG2 cells upon CYP7A1 knockdown (*N* = 3, *n* = 3). Data are one representative result with mean ± SD, analysis by two-way ANOVA. (H) Representation of animal experiment methodology in C57Bl/6 male mice, 10 animals per cohort were taken, and bacterial organ burden in ileum, cecum, and feces in mice was determined (*N* = 2, *n* > 5), analysis by Mann-Whitney test; *P* values: ****<0.0001, ***<0.001, **<0.01, *<0.05.

To study the function of YqhD in *Salmonella,* we observed the *yqhD* mRNA expression in STM WT at various time points (3 h, 6 h, 9 h, and 12 h). We observed that *yqhD* expression was highest at 3 h and reduced significantly with time in LB media (Fig. S1). We used a 7% bile salt mixture to study the effect of bile in most of our experiments, as the minimal inhibitory concentration (MIC) of bile salts for *Salmonella* Typhimurium has been reported in the literature to be 7% for sodium deoxycholate and 14% for ox bile, respectively (11). On supplementing with 7% bile salts in LB media to STM WT for 3 h, *yqhD* expression decreased significantly on exposure to bile (Fig. 1A). Thus, STM downregulates *yqhD* to counteract bile stress.

To further study the role of *yqhD* in *Salmonella* Typhi and Typhimurium, a *yqhD* deleted strain was generated using the lambda red recombinase system (17). We found that the deletion of *yqhD* did not alter its *in vitro* growth in LB media (Fig. S1B and C) and when examining the effect of various stresses reported to induce *yqhD*. We did not observe a change in the growth of STM Δ*yqhD* in the presence of iron chelator 2,2′-bipyridyl and osmotic stress inducer sodium chloride compared to STM WT. STM Δ*yqhD* exhibited a higher bacterial growth in the bile salt mixture (Fig. S1D through F), implying its role in inducing bile susceptibility. STM probably reduced *yqhD* expression to survive under bile salt. We were further intrigued to investigate the role of *yqhD* in regulating the bile salt response in *Salmonella*. We observed an increase in the survival of STM Δ*yqhD* upon exposure to both primary (7% bile salts mixture) and secondary bile salt (1% sodium deoxycholate) (Fig. 1B and C). Similarly, for *Salmonella* Typhi, there was increased survival on exposure to 7% bile for STY Δ*yqhD* (Fig. 1D).

Bile is secreted by hepatocytes in the liver. Thus, we validated our observation in the hepatocyte cell line HepG2. Upon infection of STM WT, the mRNA expression of *yqhD* reduced significantly at 6 h and 16 h compared to 2 h (Fig. 1E). Upon performing a gentamicin protection assay, we observed that STM Δ*yqhD* shows a hyperproliferation compared to STM WT (Fig. 1F). Bile is produced from cholesterol in hepatocytes, with CYP7A1 (cytochrome P450 family 7 subfamily A member 1) catalyzing a rate-limiting step (18). To further strengthen our finding that bile is responsible for the hyperproliferation of STM Δ*yqhD* in the HepG2 cells, lentivirus-mediated knockdown of CYP7A1 in HepG2 cells was performed. We confirmed the knockdown using q-RT PCR and obtained 50% knockdown (Fig. S2A). We further validated it by measuring bile in the media and HepG2 cells. There was a slight reduction in the bile concentration in both the media and cells (Fig. S2B). Upon infection of CYP7A1 knockdown cells, we observed that the proliferation of STM Δ*yqhD* was similar to that of STM WT (Fig. 1G). Thus, loss of *yqhD* generates resistance to bile salts and enhances the survival of *Salmonella* in the presence of bile, and the knockdown of CYP7A1 in hepatocytes reverses the advantage. Next, we examined the effect of sodium cholate in C57BL/6 mice, one of the major primary bile salts secreted by hepatocytes in the liver (19). 8% sodium cholate treatment in mice has been reported to increase colonization of STM WT (19). Upon treatment with sodium cholate, we observed a 2-log fold increase in colonization of STM Δ*yqhD* in the cecum compared to vehicle-treated mice, whereas there was a 1-log fold increase in STM WT colonization. In feces, STM Δ*yqhD* exhibited 2.2 log fold increase compared to vehicle-treated mice, whereas STM WT showed a 2.8-fold increase (Fig. 1H). However, the STM WT burden increased by 4 log fold in the ileum, and STM Δ*yqhD* increased 2 log fold compared to their vehicle-treated mice. These results show that exposure to sodium cholate in mice leads to increased organ burden of STM Δ*yqhD* in the cecum compared to STM WT relative to their vehicle controls. Our results are consistent with the literature, which shows that a cholesterol-rich diet increases the cecum colonization of *Salmonella* Typhimurium in 129 × 1/SvJ mice (20).

**Fig 2:**
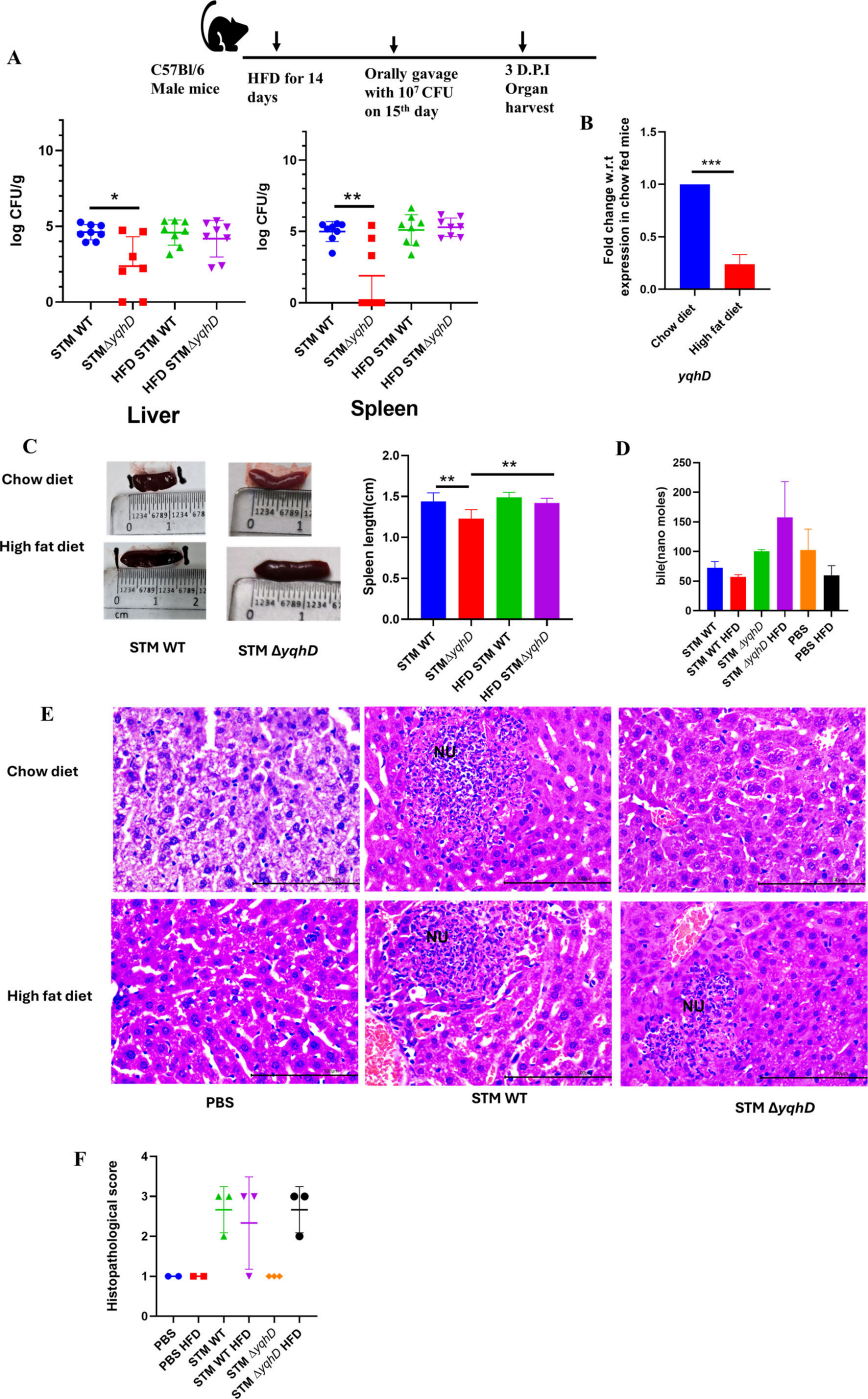
High-fat diet increases organ burden of STM Δ*yqhD* in secondary sites of infection in C57BL/6 mice. (A) Schematic of infection and bacterial organ burden in liver and spleen of STM WT and STM Δ*yqhD* in C57BL/6 male mice 3 days post-infection, eight animals per group were taken (*N* = 2, *n* > 5). Data are one representative result with mean ± SD, analysis by the Mann-Whitney test. (B) mRNA expression of *yqhD* in STM WT upon infection in C57BL/6 mice liver upon exposure to a high-fat diet compared to chow-fed mice (*N* = 3, *n* = 3). Data are one representative result with mean ± SD, analysis by unpaired Student’s *t*-test. (C) Representative spleen images and quantification of spleen length from mice infected with STM WT and STM Δ*yqhD* chow diet and high-fat diet (*N* = 2, *n* > 5). Data are one representative result with mean ± SD, analysis performed by Mann-Whitney test. (D) Measurement of bile in the serum samples of mice. (E) Representative image of hematoxylin-eosin-stained liver sections upon *Salmonella* infection at the third-day post-infection at 40×. Scale bar = 100 µm. Here, NU is neutrophil aggregation. (F) Quantification of the data in panel E. Pathology score was assigned according to histological activity index: severe—4; moderate—3; mild—2; minor/minimum—1. Three images were acquired for infected groups and two images for PBS control. Each dot in the plot represents one mouse. *P* values: ****<0.0001, ***<0.001, **<0.01, *<0.05.

Furthermore*,* bile has fat-emulsifying properties, and it helps emulsify lipids (18). Bile secretion is induced upon a high-fat diet for the absorption of fats. Studies show that a high-fat diet and bile salt secretion boost *Salmonella* gut colonization (4). To examine the effect of a high-fat diet on colonization of STM Δ*yqhD* in C57BL/6 mice, mice were kept on a high-fat diet (60%) or chow diet for 14 days. Their weight and food intake were measured (Fig. S3A). On the 15th day, the mice were infected, upon infection with STM Δ*yqhD,* there was no difference in organ burden in the primary sites of infection on exposure to HFD 3 days post-infection. Colonization of STM Δ*yqhD* was reduced on the chow diet in the liver and spleen compared to STM WT, colonization of STM Δ*yqhD* increased when mice were kept on HFD compared to the chow diet and became similar to STM WT (Fig. 2A). We also observed that the mRNA levels of *yqhD* were reduced in STM WT from liver samples of STM WT infected mice on HFD compared to the chow diet (Fig. 2B). Similarly, 5 days post-infection, there was a reduced organ burden in blood, ileum, mesenteric lymph node (MLN), liver, and spleen of STM Δ*yqhD* when mice were on a chow diet compared to STM WT. STM Δ*yqhD* organ burden significantly increased when mice were fed HFD (Fig. S4A). In line with the organ burden data, there was increased weight reduction in STM Δ*yqhD-*infected mice when kept on HFD compared to STM WT (Fig. S4B). Similar observations were validated by measuring the spleen length, where STM Δ*yqhD-*infected mice have reduced spleen length on chow diet compared to STM WT. Spleen length was significantly increased in STM Δ*yqhD* infection of mice kept on HFD and became similar to STM WT (Fig. 2C). We also measured the bile concentration in the mice serum samples. In accordance with previous literature, we observed decreased serum bile in HFD mice infected with STM WT or PBS compared to the chow diet control (21). Interestingly, we observed an increased bile concentration in STM Δ*yqhD* HFD mice compared to the chow diet control. However, the observed changes in bile salts concentration were not statistically significant (Fig. 2D). This increased circulatory bile in STM Δ*yqhD-*infected mice might be responsible for the increased organ burden of STM Δ*yqhD* in the liver and spleen on HFD compared to the chow diet. Additionally, upon hematoxylin-eosin staining, we observed that liver sections had minor pathology in the mice infected with STM Δ*yqhD* on the chow diet compared to the STM WT, which had several areas with neutrophil aggregation. Similar histopathological scores for STM WT and STM Δ*yqhD* were observed in mice on HFD with various regions of neutrophil aggregation (Fig. 2E and F; Fig. S5). Thus, treating mice with HFD leads to an increased organ burden in the liver and spleen of STM Δ*yqhD-*infected mice compared to the chow diet.

Recent reports have shown different immune responses to infection in male and female rats (22). We reproduced our experiment in female mice by shifting them to HFD for 14 days and observed a similar trend. STM Δ*yqhD* has a lesser organ burden in blood, Ileum, MLN, and liver than STM WT when kept on a chow diet, and it becomes similar to STM WT when mice are kept on HFD (Fig. S6A and B). Suggesting that increased colonization of STM Δ*yqhD* in mice is independent of the sex of the mice. In the *in vivo* pathogenesis model, the treatment of oleic acid, a primary digestive product of lard used in the HFD to mice, has been reported to induce bile synthesis (4). We observed no significant difference in the colonization of STM Δ*yqhD* compared to STM WT in the cecum, ileum, and feces during the oleic acid treatment in C57Bl/6 mice. However, the colonization of STM WT increased in the ileum on oleic acid treatment compared to vehicle control (Fig. S7).

### Oxidative stress is essential for the enhanced survival of STM Δ*yqhD* on bile salt exposure

**Fig 3:**
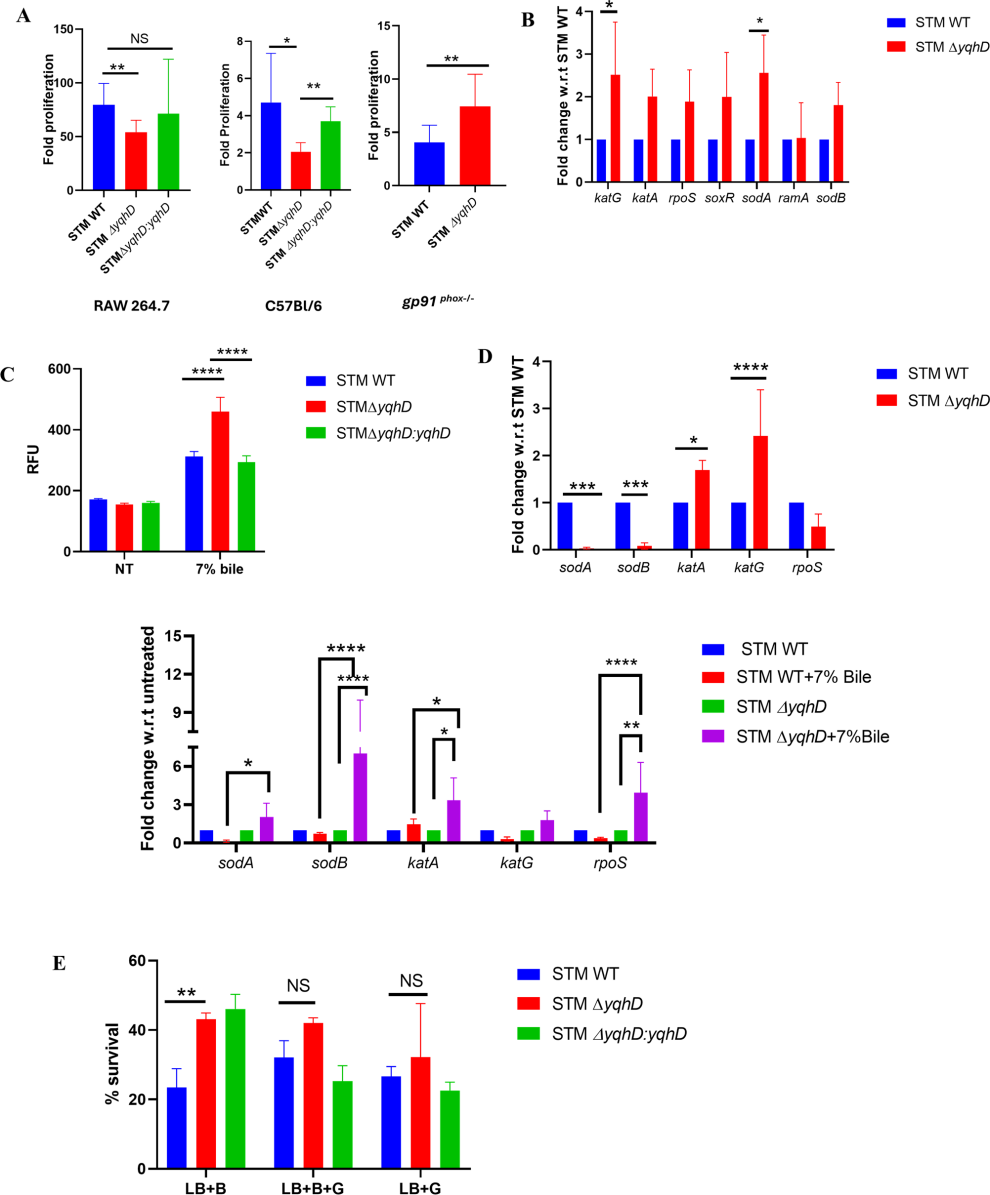
Antioxidative response of *yqhD* is essential for enhanced survival on bile salt exposure. (A) Fold proliferation in RAW 264.7 macrophages and peritoneal macrophages from C57BL/6 and *gp91 ^phox^*^−/−^ mice of STM WT, STM Δ*yqhD,* and STMΔ*yqhD:yqhD* (*N* = 3, *n* = 3). Data are one representative result with mean ± SD, analysis by unpaired Student’s *t*-test. (B) The mRNA expression of antioxidant genes in STM Δ*yqhD* in RAW 264.7 macrophages at 16 h post-infection (*N* = 3, *n* = 3). Data are one representative result with mean ± SD, analysis by two-way ANOVA. (C) DCFDA assay to measure ROS levels in STM WT, STM Δ*yqhD,* and STMΔ*yqhD:yqhD* on treatment with 7% bile (*N* = 3, *n* = 3). Data are one representative result with mean ± SD, analysis by two-way ANOVA. (D) The mRNA expression of antioxidant genes in STM Δ*yqhD* as compared to STM WT and in the presence of 7% bile in the STM Δ*yqhD* and STM WT at 3 h (*N* = 3, *n* = 3). Data are one representative result with mean ± SD, analysis by two-way ANOVA. (E) The *in-vitro* sensitivity assay on the treatment of STM Δ*yqhD* on exposure to bile salts in the presence of 10 mM glutathione by CFU/ml (*N* = 3, *n* = 2). Data are one representative result with mean ± SD, analysis by two-way ANOVA. *P* values: ****<0.0001, ***<0.001, **<0.01, *<0.05.

*yqhD* in literature has been shown to have an antioxidant function, and bile salts induce oxidative stress by producing ROS. We observed that fold proliferation of the STM Δ*yqhD* is attenuated in macrophages, as seen in our *in-vitro* study in RAW 264.7 macrophages and peritoneal macrophages from C57BL/6 mice (Fig. 3A). ROS is generated through the NADPH-phagocytic oxidase as an immediate measure to combat the invading pathogen (23). STM Δ*yqhD* proliferation was significantly higher in peritoneal macrophages from mice deficient in the NADPH phagocytic oxidase(*gp91^phox−/^*^−^) (Fig. 3A). We assessed the mRNA expression of various antioxidant genes in the RAW 264.7 macrophages 16 h post-infection. We observed that all the antioxidant genes are upregulated in STM Δ*yqhD* compared to STM WT (Fig. 3B). Thus, consistent with previous literature, *yqhD* helps maintain the redox balance. We also determined the intracellular ROS levels upon bile salt exposure using dichlorofluorescein diacetate (DCFDA). In the presence of 7% bile, STM Δ*yqhD* showed a higher intracellular ROS (Fig. 3C). Interestingly, the survival of STM Δ*yqhD* in the presence of bile salts is increased compared to WT even though it has higher ROS. In the literature, bacteria’s oxidative stress genes (catalase and superoxide dismutase) are downregulated on bile salt exposure (24).

On qRT PCR analysis of various oxidative stress genes, we observed that STM Δ*yqhD* has upregulation of *katA, katG* and downregulation of *sodA, sodB*. On bile salt exposure, *sodA, sodB*, and stress response molecule *rpoS* were significantly upregulated. Thus, *sodA* and *sodB* are induced in the STM Δ*yqhD* in response to bile (Fig. 3D). To elucidate whether the increased survival of STM Δ*yqhD* on bile salt exposure was due to oxidative stress. Antioxidant glutathione and the primary bile salt treatment were added to the LB media. We observed no significant difference in the survival of STM WT and STM Δ*yqhD*. There was increased survival of STM WT on glutathione addition to bile salts, although not significant, while survival remains similar for STM Δ*yqhD* (Fig. 3E). Taken together, these observations suggest that the reduction of oxidative stress does not increase the survival of the STM Δ*yqhD* and is advantageous to STM WT in bile salt exposure.

**Fig 4:**
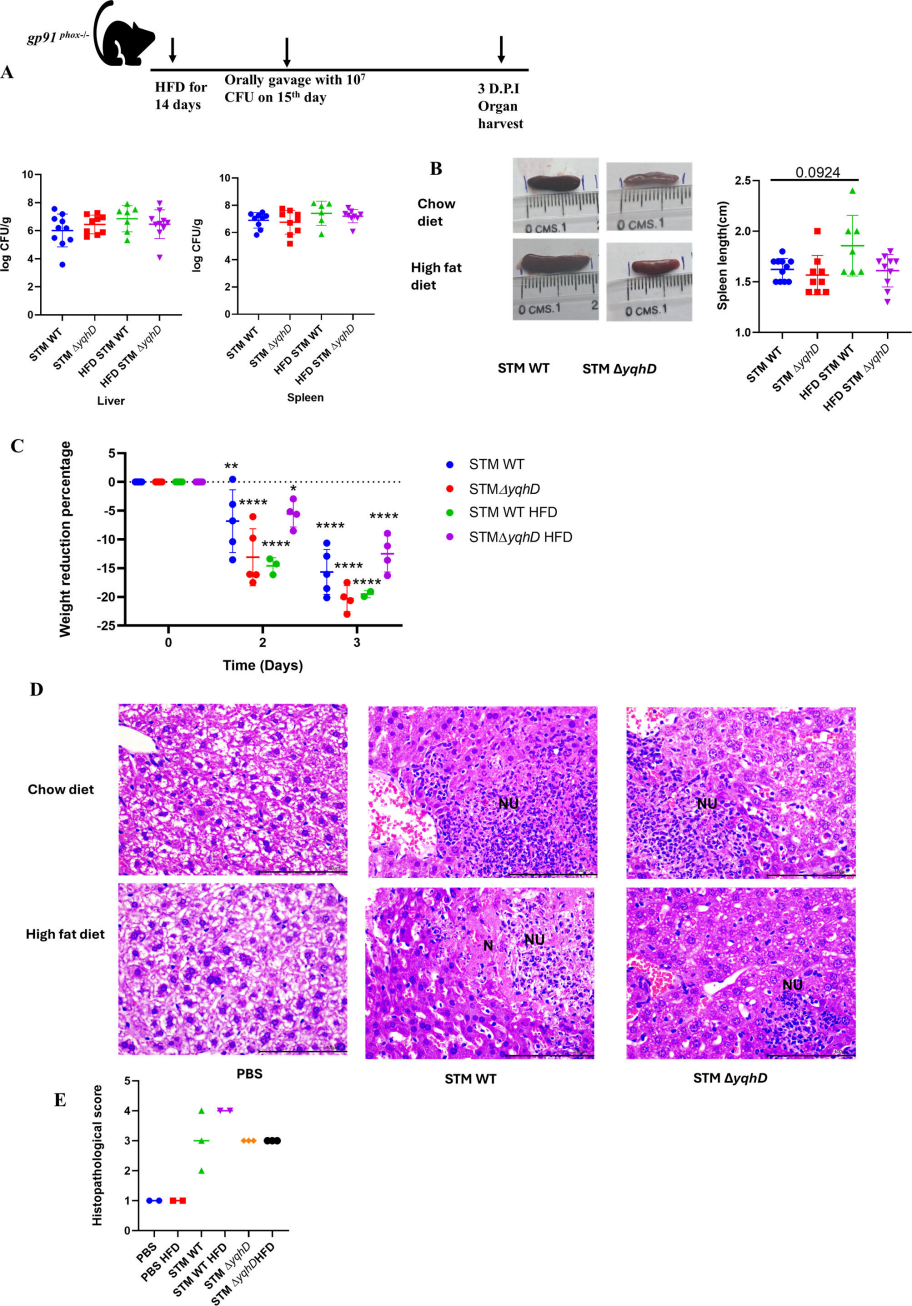
Oxidative stress is essential for increased organ burden of STM Δ*yqhD* in *gp91 ^phox−/^*^−^ mice on treatment with a High-fat diet. (A) Schematic of infection and organ burden in liver and spleen of STM WT and STM Δ *yqhD* in *gp91 ^phox−/^*^−^ male mice 3 days post-infection (five animals per group), data are presented with mean ± SD and combined from two experiments. (B) Representative spleen images and quantification of spleen length from mice infected with STM WT and STM Δ *yqhD* on chow diet and high-fat diet, data are presented with mean ± SD and combined from two experiments, analysis performed by Mann-Whitney test (*N* = 2, *n* > 5). (C) Average weight reduction on infection of STM WT and STM Δ*yqhD* in *gp91 ^phox−/^*^−^ post*^-^*infection, five animals per cohort were taken, and data are presented as mean ± SD analysis by two-way ANOVA. (D) Representative image of hematoxylin-eosin-stained liver sections upon *Salmonella* infection on the third-day post-infection at 40×. Scale bar = 100 µm. Here, NU is neutrophil aggregation, and N is necrosis. (E) Quantification of the data in panel D. Pathology score was assigned according to histological activity index as severe—4; moderate—3; mild—2; minor/minimum—1. Three images were acquired for infected groups and two images for PBS control. Each dot in the plot represents one mouse. *P* values: ****<0.0001, ***<0.001, **<0.01, *<0.05.

To determine the effect of oxidative stress and a high-fat diet, we used *gp91^phox−/^*^−^ male mice and kept them on HFD for 14 days, measured their weight and food intake (Fig. S3B), and infection was given on the 15th day. We observed that STM WT and STM Δ*yqhD* had a similar organ burden in the liver and spleen on the chow diet and HFD. STM WT showed an increase in organ burden on HFD compared to chow, but it was not significant (Fig. 4A). Spleen length was similar in mice infected with STM WT and STM Δ*yqhD* on the chow diet. However, there was a significant increase in spleen length of mice infected with STM WT on HFD compared to STM WT on chow diet (Fig. 4B). On the analysis of the mice weight post-infection, there was an increased weight reduction in the mice infected with STM WT on HFD as compared to STM Δ*yqhD* (Fig. 4C). Upon hematoxylin and eosin staining of the liver samples from these mice, we observed that there was similar pathology in both STM WT and STM Δ*yqhD* on chow diet with neutrophil aggregation. While on HFD, there was severe pathology with STM WT infection having necrosis and neutrophil aggregation as compared to STM Δ*yqhD* (Fig. 4D and E; Fig. S8). In conclusion, STM Δ*yqhD* requires oxidative stress for the increased organ burden on HFD compared to a chow diet, as seen in the C57BL/6 mice; removal of oxidative stress in *gp91 ^phox−/^*^−^ mice increases the pathogenesis of STM WT, leading to more pathology in the liver and enhanced spleen length while providing no advantage to STM Δ*yqhD*.

### *yqhD* reduced the survival of *Salmonella* to bile by regulating the efflux pump activity

**Fig 5:**
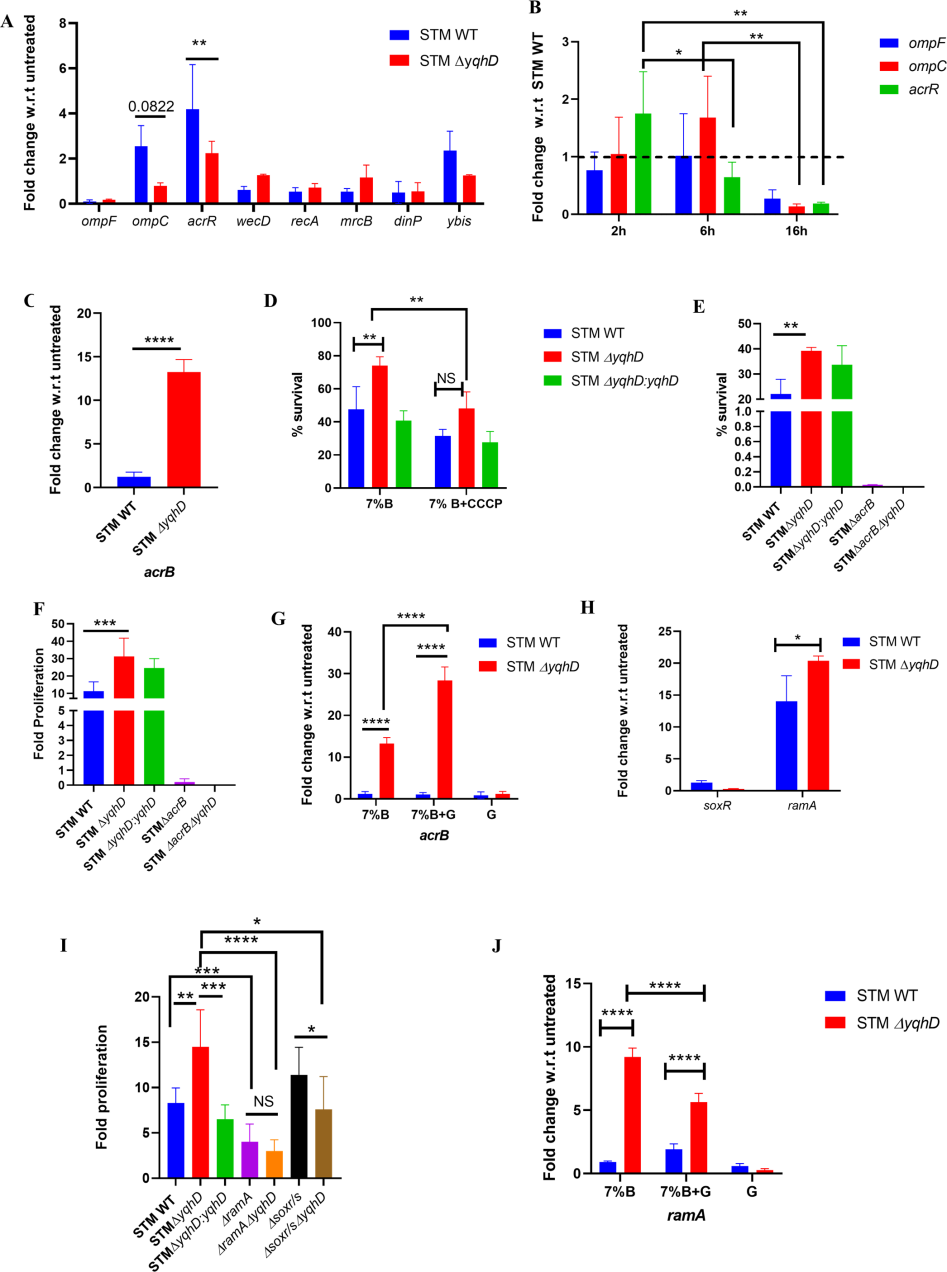
Deletion of *yqhD* downregulates AcrAB efflux pump repressor using RamA/R regulon. (A) The mRNA expression of various bile-responsive genes in STM WT, STMΔ*yqhD* on exposure to bile salts in LB media compared to untreated control at 3 h (*N* = 3, *n* = 3), data are one representative result as mean ± SD, analysis by two-way ANOVA. (B) The mRNA expression of porins and efflux pump repressor in STMΔ*yqhD* infection in HepG2 cells compared to STM WT using qRT-PCR (*N* = 3, *n* = 3), data are one representative result as mean ± SD, analysis by two-way ANOVA. (C) The mRNA expression of the *acrB* gene in STM WT, STMΔ*yqhD* on exposure to bile salts in LB media compared to untreated control at 3 h using q-RT PCR (*N* = 4, *n* = 3). Data are one representative result with mean ± SD; analysis was performed using unpaired Student’s *t*-test. (D) The *in-vitro* sensitivity assay on treatment t of 7% bile salts to STM WT, STM Δ*yqhD,* STM Δ*yqhD:yqhD* in the presence and absence of 20 µM CCCP (*N* = 3, *n* = 2). Data are one representative result with mean ± SD, analysis by two-way ANOVA. (E)The *in vitro* sensitivity assay on treatment of 7% bile salts to STM WT, STM Δ*yqhD,* STM Δ*yqhD:yqhD,* STM Δ*acrB,* STM Δ*acrB* Δ*yqhD* (*N* = 3, *n* = 2) in LB media. Data are one representative result with mean ± SD, analysis by unpaired student’s *t*-test. (F) Fold proliferation of STM WT, STMΔ*yqhD,* and STMΔ*yqhD:yqhD,* STM Δ*acrB,* STM Δ*acrB* Δ*yqhD* in HepG2 cells (*N* = 3, *n* = 3). Data are one representative result with mean ± SD; analysis performed using unpaired Student’s *t*-test. (G) The mRNA expression of *acrB* in STM WT and STM Δ*yqhD* on treatment with bile salts, glutathione alone, or in combination (*N* = 3, *n* = 3) at 3 h. Data are one representative result with mean ± SD, analysis by two-way ANOVA. (H) The mRNA expression of *soxR and ramA* in STM Δ*yqhD* and STM WT on treatment with bile salts in LB media compared to untreated control at 3 h using qRT PCR (*N* = 3, *n* = 3). Data are one representative result with mean ± SD, analysis by two-way ANOVA. (I) Fold proliferation of STM WT, STMΔ*yqhD* and STMΔ*yqhD:yqhD,* STM Δ*ramA,* STM Δ*ramA*Δ*yqhD,* STM Δ*soxR/S,* STM Δ*soxR/S*Δ*yqhD* in HepG2 cells (*N* = 3, *n* = 3). Data are one representative result with mean ± SD, analysis by unpaired Student’s *t*-test. (J) The mRNA expression of *ramA* in STM WT and STM Δ*yqhD* on treatment with bile salts, glutathione alone, or in combination (*N* = 3, *n* = 3) at 3 h. Data are one representative result with mean ± SD, analysis by two-way ANOVA. *P* values: ****<0.0001, ***<0.001, **<0.01, *<0.05.

To elucidate the mechanism behind the increased survival of the *yqhD* knockout, we performed qRT-PCR analysis for various bile stress genes—porins (*ompF, ompC),* acrAB efflux pump repressor (*acrR),* cell envelope (*wecD, mcrB, ybis*), and DNA damage (*recA, dinP*) reported in the literature for bile salt resistance in enteric bacteria. We observed a significant decrease in mRNA expression of the efflux pump repressor (*acrR*), and *ompC* was decreased but not significantly in the *yqhD* mutant upon treatment with bile salts compared to STM WT (Fig. 5A). Similarly, upon infection into the HepG2 cells, the *yqhD* mutant exhibited a significant time-dependent decrease in the mRNA levels of efflux pump repressor (*acrR*) at 6 h and 16 h, *ompC* at 16 h compared to 2 h (Fig. 5B). The efflux pump repressor (*acrR*) downregulation was consistent in both *in vitro* and in HepG2 cells indicating increased activity of AcrAB efflux pump. On analysis of a key component of AcrAB efflux pump gene, *acrB* (located in the inner membrane of *Salmonella),* STM Δ*yqhD* showed higher upregulation as compared to STM WT on bile salt treatment (Fig. 5C). Furthermore, on treatment with the efflux pump inhibitor carbonyl cyanide m-chlorophenylhydrazone (CCCP) along with the bile salt treatment, survival of both STM WT and STM Δ*yqhD* was reduced, but it was much more significant in STM Δ*yqhD* (Fig. 5D). To further strengthen the observation that the efflux pump activity mediates the enhanced survival of the *yqhD* mutant, we generated an *acrB* mutant in the background of *yqhD* deletion. The STM Δ*acrB* Δ*yqhD* showed no growth upon bile salt exposure compared to STM Δ*acrB,* which showed highly attenuated growth (Fig. 5E). Thus, the growth advantage of STM Δ*yqhD* under bile stress is lost upon deletion of both *acrB* and *yqhD*. Similarly, we observed a reduction in fold proliferation of STM Δ*acrB* Δ*yqhD* compared to STM Δ*yqhD* and STM Δ*acrB* upon infection into the HepG2 cells (Fig. 5F). The above results suggest that *yqhD* mediates sensitivity to bile by regulating the AcrAB efflux pump activity.

Therefore, an interesting question arises: how does cytosolic protein YqhD regulate the AcrAB efflux pump activity? We hypothesized that elevated oxidative stress upon the deletion of *yqhD* leads to the activation of efflux pump activity. To determine the effect of ROS on *acrB* induction in STM Δ*yqhD,* mRNA expression of *acrB* was measured by adding glutathione and bile salts together or individually. STM Δ*yqhD* showed upregulation of *acrB* expression in both conditions—on bile salt treatment alone or in combination with glutathione (Fig. 5G). Thus, increased *acrB* expression is the primary effect of *yqhD* deletion.

### *yqhD* regulates the efflux pump activity using the RamA/R two-component system

The AcrAB efflux pump is regulated majorly by *ramA* in *Salmonella*, and *soxR/S* plays a small role in induction (25). Upon treatment with bile salts, we assessed the mRNA levels of both *ramA* and *soxR* and observed a significant increase in *ramA* and a decrease in *soxR* expression in the *yqhD* mutant compared to STM WT (Fig. 5H). We further generated deletion mutants for *ramA* and the *soxR/S* regulon in the background of the *yqhD* mutant. We observed that STM Δ*ramA*Δ*yqhD* showed reduced survival upon exposure to 7% bile salts as compared to STM Δ*yqhD* and STM Δ*ramA,* as measured by spotting on an LB agar plate supplemented with 7% bile salt (Fig. S9). Similarly, in HepG2 cells, the proliferation of the *yqhD* mutant was significantly reduced when *ramA* was mutated. The single deletion of *ramA* has also attenuated the proliferation in HepG2 cells compared to STM WT (Fig. 5I).Thus, the RamA/R regulon mediates the increased survival of STM Δ*yqhD* on bile salt exposure. To determine whether the increased *ramA* expression is due to the deletion of *yqhD* or enhanced reactive oxygen species (ROS) generated in STM Δ*yqhD* on bile salt exposure. Upon performing q-RT PCR analysis, we observed that with the treatment of antioxidant glutathione with bile salts, *ramA* expression levels were higher in STMΔ*yqhD* than in STM WT in both conditions (bile salt with and without glutathione). On addition of glutathione along with bile salts, *ramA* expression was significantly reduced in STMΔ*yqhD* compared to only bile salt treatment (Fig. 5J). The induction of *ramA* in STM Δ*yqhD* seems to be partially dependent on the increased ROS generated during bile salt exposure. These observations lead us to conclude that RamA/R regulon mediates the increased survival of STM Δ*yqhD* upon bile salts exposure, HepG2 cells and is mediated partially by oxidative stress.

### Bile stress does not reduce invasion in STM Δ*yqhD*

Our study suggests that the absence of YqhD in *Salmonella* is advantageous for its survival in the presence of bile. Thus, we wanted to question how *Salmonella* utilizes YqhD to its advantage. While the invasion into the colon carcinoma cells (Caco-2) was similar in STM WT and STM Δ*yqhD* (Fig. S10A). Interestingly, we observed that the invasion in hepatocytes (HepG2) was reduced significantly in STMΔ*yqhD* as compared to STM WT (Fig. S10B). These results indicate that the invasion of the mutant is altered in the HepG2, unlike Caco-2 cells. We hypothesized that the reduction of invasion in HepG2 cells could be due to the downregulation of SPI-1 machinery by bile salt produced by hepatocytes.

*Salmonella* invasion in eukaryotic cells is mediated by the type three secretion system 1 encoded by *Salmonella* pathogenicity island 1 (SPI-1). Bile is an environmental cue to repress SPI-1 genes by destabilizing the HilD (26–28). To understand whether the reduced invasion of STM Δ*yqhD* is due to the modulatory function of bile in the expression of SPI-1 genes, we treated STM WT and STM Δ*yqhD* with 7% bile salt in LB media for 6 h. It was observed that SPI-1 encoded genes (*invF* and *sipA*) were significantly downregulated in STM WT on bile salt treatment. At the same time, in STM Δ*yqhD,* SPI-1 expression was reduced on bile salt exposure. However, it was still higher than STM WT (Fig. S10C). This observation that the STM Δ*yqhD* mutant exhibited higher SPI-1 expression compared to the STM WT on bile salt exposure, yet demonstrated decreased invasion of HepG2 cells, suggests that factors beyond bile-mediated repression of SPI-1 expression levels influence invasion efficiency of STM Δ*yqhD*. Thus, to confirm our results further, we observed the invasion with the knockdown of the CYP7A1 in HepG2 cells. Invasion of STM Δ*yqhD* was attenuated even in knockdown conditions compared to STM WT (Fig. S10D). Thus, our results suggest that bile secretion is not responsible for the reduced invasion of STM Δ*yqhD* in the HepG2 cells. There might be other factors that are responsible for the observed reduced invasion of STM Δ*yqhD* in HepG2, which require further investigation.

## DISCUSSION

This study examines the growth of *Salmonella* Typhimurium 14028S and Typhi CT18 on exposure to a bile salts mixture *in vitro* and liver carcinoma (HepG2 cells), as the liver is responsible for bile production. Despite extensive research, new genes involved in bile resistance continue to be identified. Our study addresses the novel interaction between the oxidative stress gene *yqhD* and bile salts. YqhD is studied widely in *E. coli* for its antioxidant function and importance in biofuel production. In addition to its role as an antioxidant, we found that *yqhD* increased susceptibility to bile in both *in-vitro* and HepG2 cells. Treatment of mice with sodium cholate and HFD led to increased colonization by STM Δ*yqhD* (Fig. 1).

In accordance with the antioxidant function of *yqhD*, reduced fold proliferation was observed in the RAW 264.7 macrophages and peritoneal macrophages from C57BL/6 mice (Fig. 3A). Macrophages are essential for the lifecycle of *Salmonella* in the host. During the infection cycle of *Salmonella*, the bacteria reside in the macrophages and utilize the immune cells to disseminate to secondary sites of infection (29). Macrophages present a hostile environment to the pathogen by generating ROS, RNS (reactive nitrogen species), antimicrobial peptides, and nutrient deprivation. The attenuated proliferation of STMΔ*yqhD* in macrophages might account for the reduction in organ burden in C57BL/6 mice. On infection of peritoneal macrophages from NADPH-deficient *gp91* ^phox−/−^ mice, higher fold proliferation was observed in STM Δ*yqhD* compared to STM WT. The organ burden of STM WT and STM Δ*yqhD* also became similar in *gp91* ^phox−/−^ mice on the chow diet (Fig. 4A).

The increased survival advantage of the Δ*yqhD* was due to increased oxidative stress on exposure to bile salts, and treatment with glutathione *in vitro* leads to no advantage in the growth of STM Δ*yqhD* on bile treatment. In contrast, the survival of STM WT increased. Similarly, in *gp91^phox−/^*^−^ mice, there was no increase in organ burden or pathology of STM Δ*yqhD* on shifting mice to HFD compared to the chow diet. In the STM WT infection, there was a slight increase in organ burden, higher splenomegaly and pathology in the liver on HFD compared to the chow diet was observed.

Upon exposure to bile, *Salmonella* is known to modulate its envelope structures like lipopolysaccharides, peptidoglycan (*ybiS*), and the enterobacterial common antigen (*wecD*) to provide a barrier against the bile salt uptake (7, 30–32). *Salmonella* also downregulates porins (*ompF* and *ompC*) to reduce the uptake of bile salts (9). Furthermore, the efflux systems (*acrAB*) are induced, which decreases the intracellular concentration of bile salts (10, 33). Exposure to bile also upregulates the DNA damage genes (*recA* and *dinP*) to overcome the damage caused by bile using homologous recombination and SOS-associated DNA repair (11). *yqhD* was found to induce the activity of the AcrAB efflux pump, and treatment with efflux pump inhibitor CCCP or deleting the *acrB* gene of the efflux pump led to reduced survival of STMΔ*yqhD* on bile salt exposure. AcrAB efflux pump provides resistance against decanoate, bile salts, and ethanol (34). AcrAB efflux pump has also been reported for its role in colonization, persistence, and invasion and is induced by RamA in response to bile stress (35–37). The AcrAB efflux pump is regulated by the Mar operon in *E. coli*, while *ramA* is the major regulator of *Salmonella*, and *soxR/S* plays a small role in the induction (25). Paraquat, a superoxide generator, could induce the *acrAB* efflux pump using SoxS, independent of RamA (38). RamA acts on the *acrAB* efflux pump by binding to bile salts (cholic acid), where RamA is transcriptionally repressed by RamR (36, 37, 39). RamA was also reported to bind directly to alcohol dehydrogenase P(*aldhP*) in *Klebsiella pneumoniae* (40). This study found that *ramA* was induced in the STM Δ*yqhD* on bile salt more significantly than in STM WT, and its induction was partially dependent on oxidative stress. The deletion of *ramA* also inhibits the growth advantage of STM Δ*yqhD* in HepG2 cells, a similar effect as AcrAB efflux pump deletion. The above results conclude that YqhD modulated efflux pump activity using RamA/R regulon.

The current study has limitations regarding how exactly *yqhD* modulates the RamA/R regulon. Despite the improved survival on deletion of *yqhD* in the presence of bile salt, there is a significant reduction in the invasion of HepG2 cells. STM Δ*yqhD* survival on bile salt exposure was found to be mediated by RamA/R regulon, which also can mediate the invasion of *Salmonella* (41). However, we observed that knockdown of the bile salt synthesis could not rescue the invasion, thus suggesting the invasion of STM Δ*yqhD* is independent of bile. The mechanism of reduced invasion of STM Δ*yqhD* in hepatocytes remains to be elucidated.

## MATERIALS AND METHODS

### Bacterial culture

*Salmonella enterica* serovar Typhimurium 14028S strain used in the study was a kind gift from Prof. Michael Hensel. The bacterial strains were revived from glycerol stocks at −80°C and streaked on the LB agar plates with the appropriate antibiotics. All the antibiotics used in the study are from SRL. For most bacterial cultures, a single colony was taken and inoculated in an LB tube and grown at 37°C, 170 rpm, in a shaking incubator. STM pKD46 was grown at 30°C, 170 rpm in the shaking incubator. The growth curve was performed by subculturing (1:100) the overnight culture in fresh LB media, and growth was monitored for 24 h using Bioscreen. All the strains used in this study are listed in the Table S1.

### Knockout and complement generation

Knockout strains were made using the one-step gene inactivation strategy used by Datsenko and Wanner (16). STM WT with pKD46 plasmid, having lambda red recombinase system under arabinose inducible promoter, was grown in LB media supplemented with 50 µg/mL ampicillin and 50 mM arabinose at 30°C. Bacterial culture was sub-cultured (1:33) and incubated for 3 h to achieve log-phase culture. Electrocompetent STM pKD46 cells were prepared by washing the bacterial cells with Milli Q water and 10% glycerol. Kanamycin and chloramphenicol resistance gene cassettes amplified from pKD4 and pKD3 using gene-specific knockout primers were used for the electroporation. The transformed bacteria were selected on LB agar with appropriate antibiotics. Transformed bacterial colonies were patched on the fresh antibiotic plate and confirmed with gene-specific confirmatory primers.

#### Construction of the complement strain

*yqhD* gene was amplified by cloning primers using colony PCR. The PCR product and pQE60 plasmid were digested with restriction enzymes—EcoRI (NEB) and HindIII (NEB) in a cut smart buffer for 2 h at 37°C. Digested insert (PCR product) and vector (pQE-60) were gel-purified and ligated by T4 DNA ligase (NEB) in 10× ligation buffer overnight at 16°C. The recombinant plasmid was transformed into the STM Δ*yqhD*. The recombinant plasmid was further confirmed by restriction digestion.

#### Construction of double knockout strains

Single knockout strains of *ramA*, *soxr/s,* and *acrB* were transformed with pKD46 plasmid. Transformed bacteria were grown with ampicillin (50 µg/mL), chloramphenicol (25 µg/mL), and arabinose (50 mM) to log phase culture. The kanamycin resistance gene cassette amplified from the pKD4 plasmid was electroporated. The double knockout strains were selected on an LB agar plate with kanamycin (50 µg/mL) and chloramphenicol (25 µg/mL). Confirmatory primers further confirmed double knockouts. All the primers used in the study are listed in the Table S2.

### Oxidative stress experiments

Reactive oxygen species (ROS) in the bacteria were measured using redox-sensitive probe 2′,7′ dichlorodihydrofluorescein diacetate (H_2_DCFDA-Sigma-Aldrich). H_2_DCFDA, once internalized, is oxidized by intracellular esterase and converted to fluorescent 2′,7′-dichlorofluorescein (H_2_DCF) with excitation at 495 nm and emission at 529 nm. Bacteria OD was adjusted to 0.3, subjected to bile stress overnight in LB, and incubated with 10 µM of DCFDA for 30 min. Fluorescence was measured in a Tecan plate reader.

### Survival on bile exposure

Log phase culture of the bacteria was adjusted to 0.3 OD in 1 mL of phosphate buffer saline (PBS). Approximately 10^5^ bacteria were transferred to various conditions—7% of bile salt mixture (Himedia), 1% sodium deoxycholate (Sigma-Aldrich), 10 mM of glutathione (Sigma-Aldrich), 20 µM of carbonyl cyanide *m*-chlorophenylhydrazone (Sigma-Aldrich) in Luria Broth alone, or combinations prepared with a total volume of 1 mL. Treated bacteria (100 µL) were transferred to 96-well plates and incubated for 18 h in the shaking incubator at 170 rpm, 37°C. Post-incubation, absorbance was measured in a Tecan plate reader at 595 nm, and CFU analysis was performed by plating on LB agar. Percentage survival was calculated by dividing the bacteria treated in different conditions to untreated bacteria and multiplying by 100.

### Q-RT PCR analysis: RNA-cDNA from bacteria and cell culture

The bacterial cells were pelleted down at 6,000 rpm for 10 min. Pellets were lysed with TRIzol reagent (RNAiso plus-Takara) and stored at −80°C overnight. The lysed supernatant was subjected to chloroform extraction and precipitation with an equal volume of isopropanol. The precipitated RNA was air-dried and dissolved in DEPC-treated water. RNA concentration was checked using nanodrop, and the quality of the RNA was assessed through 1% agarose gel electrophoresis. cDNA was prepared post-DNase treatment (Thermo Fisher Scientific) using a PrimeScript RT reagent kit from Takara. Q-RT PCR was done using TB green (Takara) in the Bio-Rad real-time detection system. 16S rRNA was used as a housekeeping control, and dct was calculated by subtracting the specific primer ct from that of 16S. ddct were calculated by subtracting the dct of 7% bile salt treated sample with the untreated sample for the *in vitro* samples, and for HepG2 cells, the ddct was calculated by subtracting the dct at 6 h, 16h, to 2 h of STM Δ*yqhD* to that of STM WT.

Fold change = 2^−ddct^

### Cell culture

RAW 264.7 and HepG2 cells were maintained in DMEM (Lonza) supplemented with 10% FBS (Gibco) and 1% penicillin-streptomycin (Sigma-Aldrich). Peritoneal macrophages were maintained in RPMI (Lonza) with 10% FBS and 1% penicillin-streptomycin (Sigma-Aldrich). Colon carcinoma (Caco2) cells were maintained in DMEM with 10% FBS, 1% penicillin-streptomycin (Sigma-Aldrich), 1% sodium pyruvate (Sigma-Aldrich), and non-essential amino acids (Sigma-Aldrich). All cells were cultured at 37°C in 5% CO_2_.

### Isolation of peritoneal macrophages

Peritoneal macrophages were isolated from C57BL/6 mice and *gp91^phox−/−^* mice using a previously standardized protocol (42, 43). The mice were intraperitoneally injected with 8% brewer’s thioglycolate (from HiMedia). Five days post-injection, the primary macrophages were isolated from the peritoneal cavity. Any residual erythrocytes were lysed using an RBC lysis buffer (Sigma-R7757), and the isolated cells were maintained in an RPMI 1640 medium with 10% FBS and 1% penicillin-streptomycin for further experiments.

### Virus-mediated knockdown of CYP7A1 in HepG2 cells

shRNA constructs with puromycin as a selection marker was a kind gift from Professor Subba Rao. HEK 293T cells (0.3 million) were seeded in a poly-lysine coated 12 well plate to generate lentivirus. A plasmid mixture containing psPAX2, pMD2.G, and shRNA plasmid in the ratio of 4:1:4 was incubated in Opti-MEM (Gibco) for 5 min and then incubated further with the transfection reagent PEI for 15 min at room temperature. The above mixture was added to HEK cells with 350 µL of Opti-MEM. After 5 h, the complete medium was added. The supernatant containing viral titer from the cells was collected after 46 and 72 h post-transfection. The collected supernatant was centrifuged at 5,000 rpm for 10 min and filtered using a 0.45 µm filter. Six hundred microliters of filtrate was added with 1 mL of fresh medium and 1 µg/mL of polybrene to previously seeded HepG2 cells in six well plates. Cells were incubated for 48 h, and fresh, complete medium was given for 12 h. This was followed by puromycin selection every 12 h until the untransduced control cells died. The cells were maintained in media containing 1 µg/mL puromycin. Lentivirus was prepared following a previously described protocol (44). shRNA1 is CCGGCCACAGTTAATGCACTTAGATCTCGAGATCTAAGTGCATTAACTGTGGTTTTTG, and shRNA2 is CCGGCCACCTCTAGAGAATGGATTACTCGAGTAATCCATTCTCTAGAGGTGGTTTTTG.

Knockdown was confirmed by q-RT PCR, using human 18S primer as control and by measuring the bile salt concentration in the supernatant and HepG2 cells.

### Intracellular survival assay

0.2 million RAW 264.7 were infected with stationary phase culture of the different bacterial strains. The multiplicity of infection (MOI) used for the RAW 264.7 was 10. HepG2 cells were infected with a log-phase culture and an MOI of 10. For RNA isolation from the HepG2 cells, 1 million cells were infected with an MOI of 25 and lysed with Trizol at different time points.

Briefly after the infection with bacteria, the cells were centrifuged for 5 min at 800 rpm, followed by incubation at 37°C in 5% CO_2_ for 25 min. Cells were washed with 1× PBS and incubated with complete media containing 100 µg/mL of gentamicin to kill the bacteria outside the eukaryotic cells for 1 h. Subsequently, cells were washed with 1× PBS and complete media containing 25 µg/mL of gentamicin was added to prevent secondary infection. Cells were lysed post 2 h of 100 µg/mL of gentamicin using 0.1% Triton X-100 to calculate percent phagocytosis and percent invasion. The bacterial number was determined by plating on LB agar. Similarly, the bacteria used in the infection was determined by plating the pre-inoculum. The ratio of bacteria at 2 h in the cell lysate to pre-inoculum, multiplied by 100 gave us per cent phagocytosis for macrophages and per cent invasion for the epithelial cells. For calculations of fold proliferation, cells were lysed at 16 and 2 h post-infection. The bacterial number was determined by plating on LB agar. Fold proliferation is the ratio of the bacterial CFU at 16 h to the bacterial CFU at 2 h.

### Animal experiments

All the animals used in this study were acquired from the central animal facility (CAF), Indian Institute of Science, Bangalore. Five- to six-week-old C57BL/6 or *gp91^phox−/^*^−^ mice were used for the *in vivo* infection and peritoneal macrophage isolation.

One hundred microliters of 10 mg/mL oleic acid (Sigma-Aldrich) and 8% sodium cholate (Sigma-Aldrich) were orally gavaged to 5- to6-week-old C57BL/6 mice 1 h before infection and 4 h after infection. Oleic acid was dissolved in DMSO, and sodium cholate was dissolved in Milli-Q water. For both compounds, appropriate vehicle controls were used. Mice were infected with 10^7^ CFU/mL (100 µL) of STM WT, STM Δ*yqhD*. Mice were euthanized 24 h post-infection, and the ileum, cecum, and feces were collected. Feces were removed from the ileum and cecum using forceps before sample collection.

Mice were maintained on a maintenance diet—1324 from Altromin for rats/mice (19.20% crude protein, 4.10% crude fat, 6.10% crude fiber, and 6.90% crude ash)—and transferred to a high-fat diet (60% from ICMR) for 14 days, and their weight was monitored every alternate day. They were infected with 10^7^ CFU/mL (100 µL) of STM WT and STM Δ*yqhD* in cohorts of five animals for *gp91^phox−/^*^−^ and eight for C57BL/6 by oral gavage on the 15th day. On the third day, post-infection mice were sacrificed, and blood, ileum, mesenteric lymph nodes (MLN), liver, and spleen were harvested for the bacterial burden, RNA, 3.5% paraformaldehyde, before sample preparation for hematoxylin and eosin staining. Blood from mice was isolated using heart puncture and added to EDTA-containing vials. Isolated organs were homogenized and plated in *Salmonella Shigella* agar. All the CFU/mL obtained were normalized to the organ weight of the sample. The composition of the high-fat diet used in the study is in Table S3.

For histopathological scoring of hepatic necroinflammation/pathology score: The comparison of pathological changes in the liver tissues was evaluated under light microscopy by a veterinary pathologist and scored using a scientific method. The hepatic necroinflammation score was done according to the histological activity index (HAI) criteria with slight modification (45). The scoring of necroinflammation is graded as 0–4 for each of the pathological lesions (severe—4; moderate—3; mild—2; minor/minimum—1; no pathology—0), considering the following pathological lesions: focal inflammation, focal necrosis, and portal inflammation/portal lymphohistiocytic. Histopathological examination was performed in a blind manner. The veterinary doctor performed the histopathological scoring of liver samples without knowing the experiment or the different cohorts of mice used.

### Bile measurement

The experiment was performed per the kit protocol (ab239702). Briefly, serum samples harvested from the mice and stored at −80°C were thawed and added to the 96-well plate. The probe and enzyme mix were added, and absorbance was measured at 405 nm.

### Spot assay

Log phase culture bacteria adjusted to 0.3 OD in 1× PBS were diluted and used to spot 5 µL on the LB agar plates with or without 7% bile salt mixture. Plates were incubated overnight at 37°C. Images were acquired using Gel Doc.

### Statistical analysis

GraphPad Prism 8.4.3 was used to perform all the statistical analysis. Analysis was done using unpaired two-tailed Student’s *t*-tests and two-way ANOVA. In the animal experiments, the Mann-Whitney *U* test was performed. Statistics tests used in different experiments are mentioned in the figure legends.

## Data Availability

All data are available in the main text or supplemental material.

## SUPPLEMENTAL MATERIAL

Supplemental material msphere.00382-25-s0001.docx Supplemental figures and tables.

ASM does not own the copyrights to Supplemental Material that may be linked to, or accessed through, an article. The authors have granted ASM a non-exclusive, world-wide license to publish the Supplemental Material files. Please contact the corresponding author directly for reuse.
