# Supplementary material for: The resilience of Salmonella to bile stress is impaired due to the reduced efflux pump activity mediated by the antioxidant enzyme YqhD
Source: mSphere. 2025 Oct 2;10(10):e00382-25. doi: 10.1128/msphere.00382-25 (PMC12570503; doi:10.1128/msphere.00382-25)
Supplement: Supplemental material — Supplemental figures and tables. [file msphere.00382-25-s0001.docx]

**Supplementary Data**

**Supplementary data legends**

**Supplementary Fig.1. Growth kinetics of the various strains used in the study and their response to various stresses**


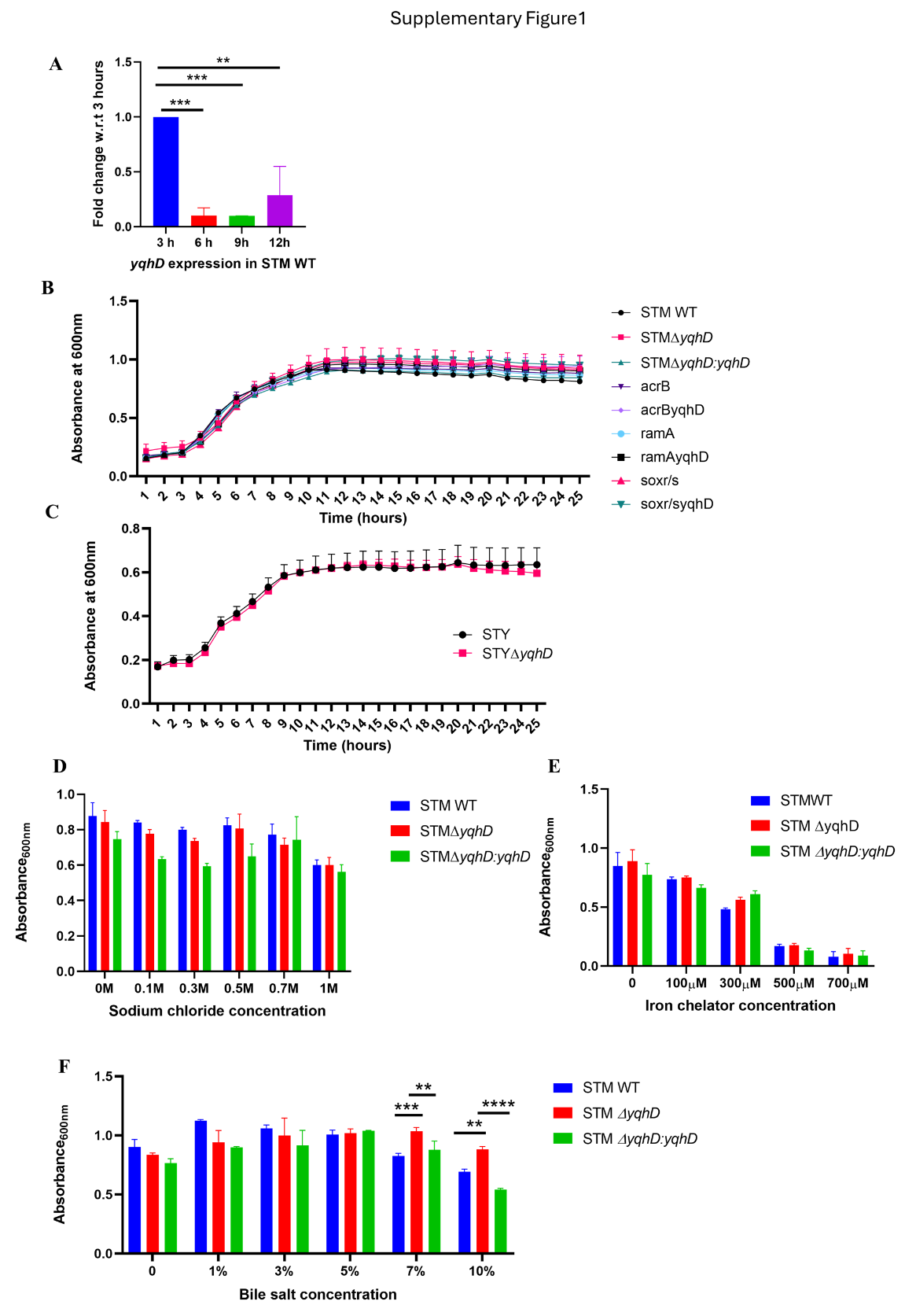


**A**. The mRNA expression of *yqhD* at different time points in LB media in STM WT with respect to 3 hours (N=3,n=3). Data are one representative result with mean ± SD, analysis was performed using one-way ANOVA **B, C**. Growth kinetics of various strains used in the study(N=3,n=5). Data are one representative result with mean ± SD. **D**. Absorbance at various concentrations of sodium chloride (N=3,n=3). Data are one representative result with mean ± SD. **E**. Absorbance at various concentrations of iron chelator 2,2′-bipyridine.(N=3,n=3). Data are one representative result with mean ± SD. **F**. Absorbance at various concentrations of bile salts (N=3,n=3). Data are one representative result with mean ± SD, analysis was performed using two-way ANOVA; p values****<0.0001, ***<0.001, **<0.01, *<0.05.

**Supplementary Fig.2.- Knockdown of CYP7A1 in HepG2 cells**


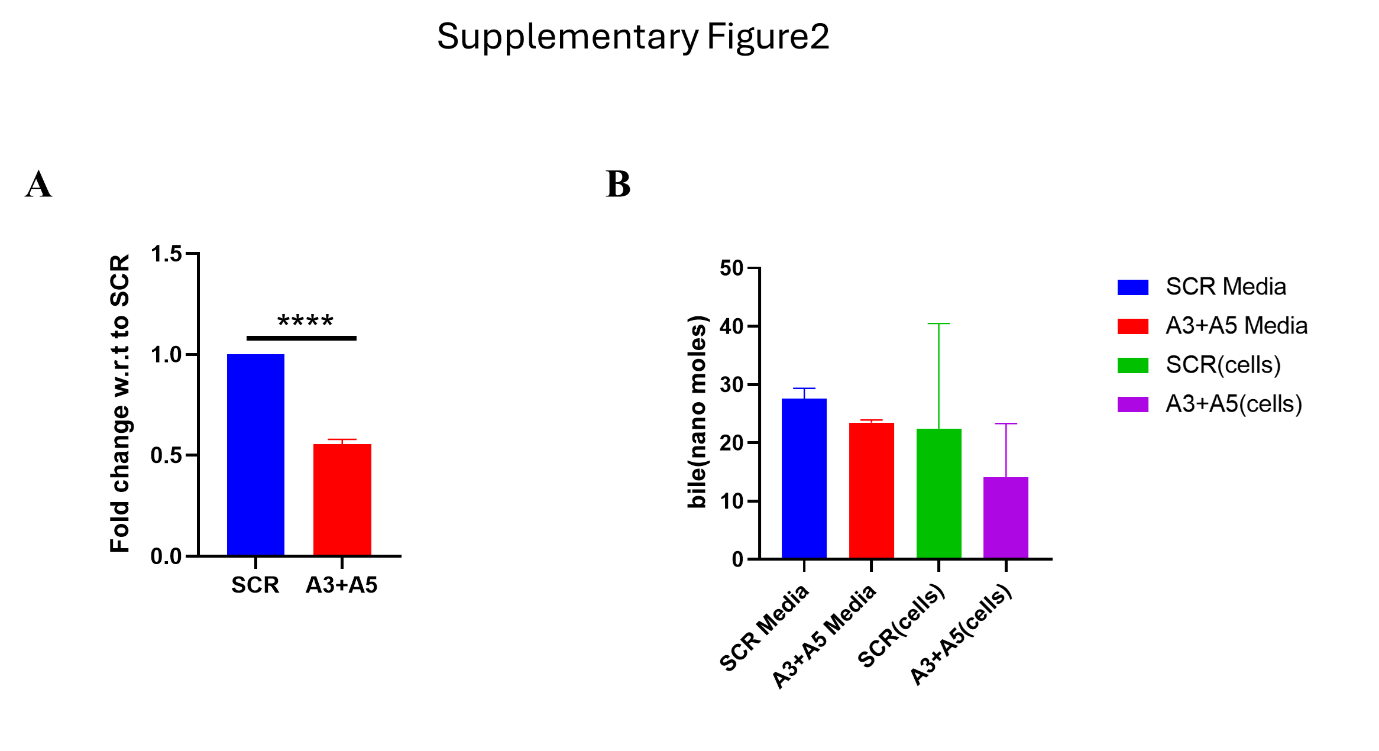


**A**. Validation of knockdown of CYP7A1 in HepG2 cells using qRT PCR(n=3). Data are representative result with mean ± SD. Analysis was performed using an unpaired two-tailed Student's t-test. p values****<0.0001, ***<0.001, **<0.01, *<0.05. **B.** Bile estimation upon knockdown in HepG2 cells (n=3).

**Supplementary Fig.3. Average weight of mice fed on chow and high-fat diets.**


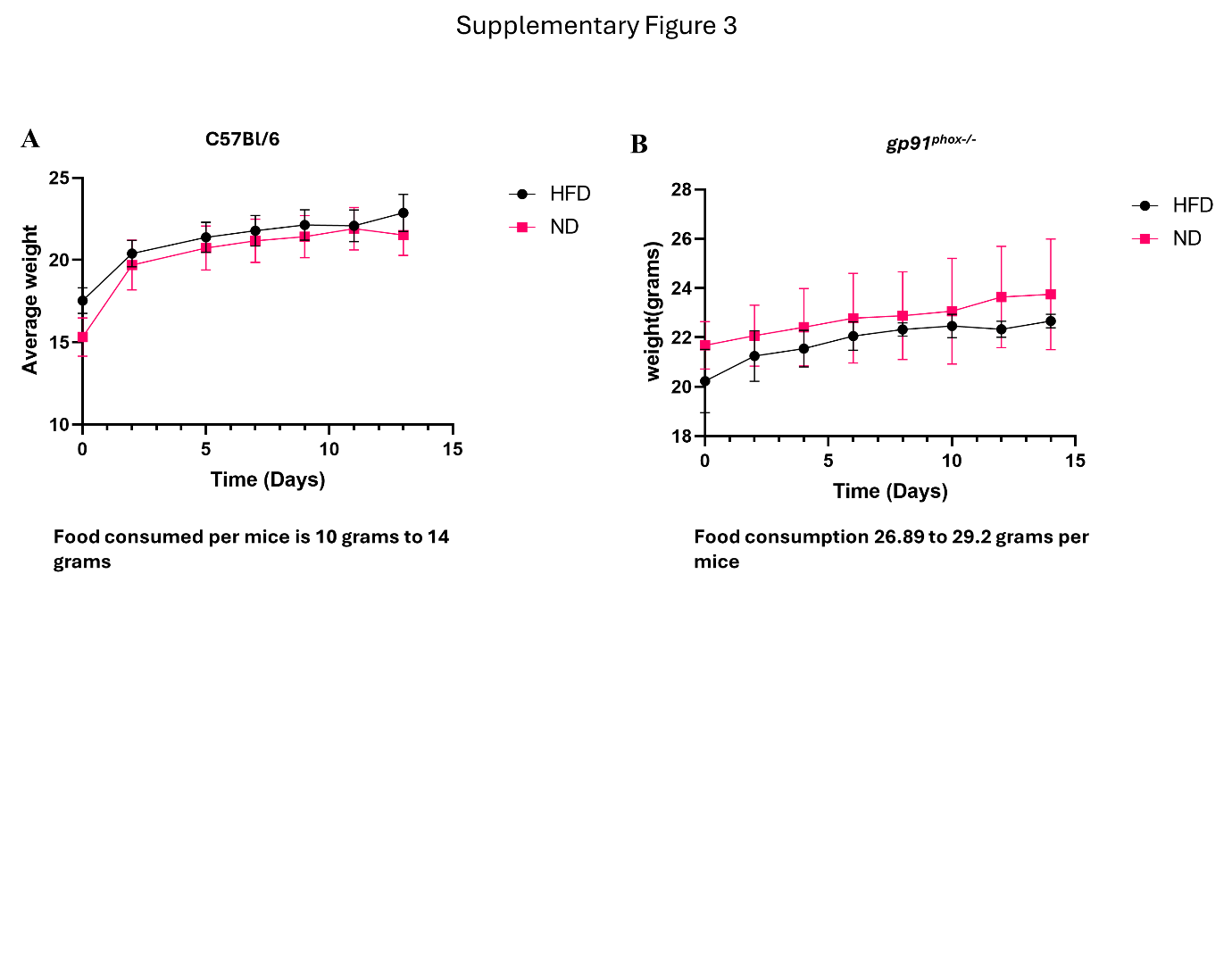


1. Average weight of mice on treatment with different diets for 15 days ( HFD- high fat diet, ND- chow diet).
2. Average weight of *gp91* ^phox-/-^ mice on treatment with different diet for 15 days ( HFD- high fat diet, ND- chow diet).

**Supplementary Fig.4. High-fat diet increases organ burden of STM Δ*yqhD* in C57BL/6 male mice at 5 days post-infection.**


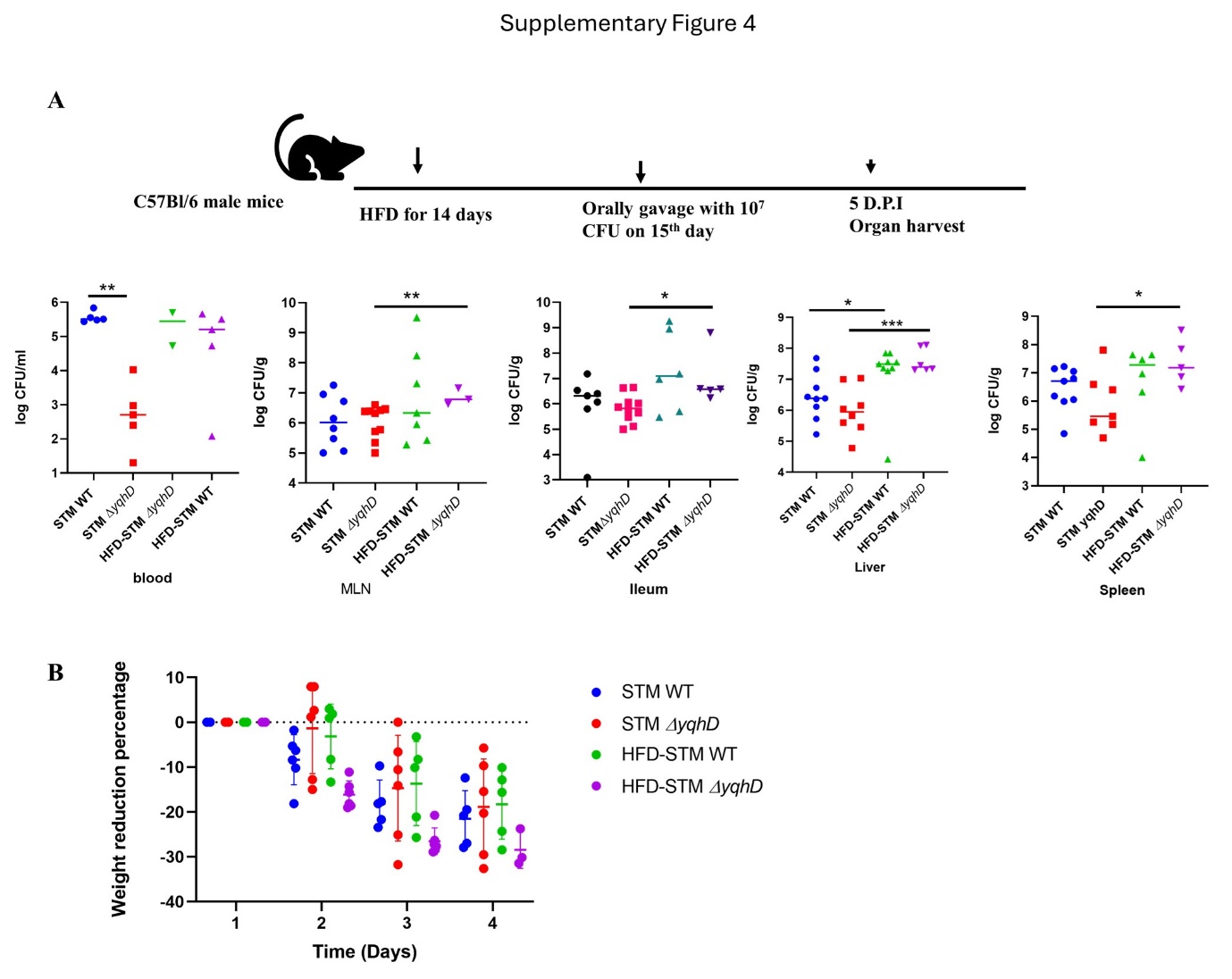


1. Schematic of infection and organ burden of STM WT and STM Δ *yqhD* in C57BL/6 male mice at 5 days post-infection, five animals per cohort were taken, data are presented with mean ± SD, analysis performed by Mann- Whitney test: p values****<0.0001, ***<0.001, **<0.01, *<0.05. Both the dilutions have been used for plotting the data as there was mortality in STM *ΔyqhD* on the HFD treatment.
2. Weight reduction after infection with various strains with respect to uninfected in the same group, five animals per cohort were taken, data are presented with mean ± SD.

**Supplementary Fig.5. Haematoxylin and eosin staining of the liver sections from C57BL/6 mice at various magnifications with the pathology score**.


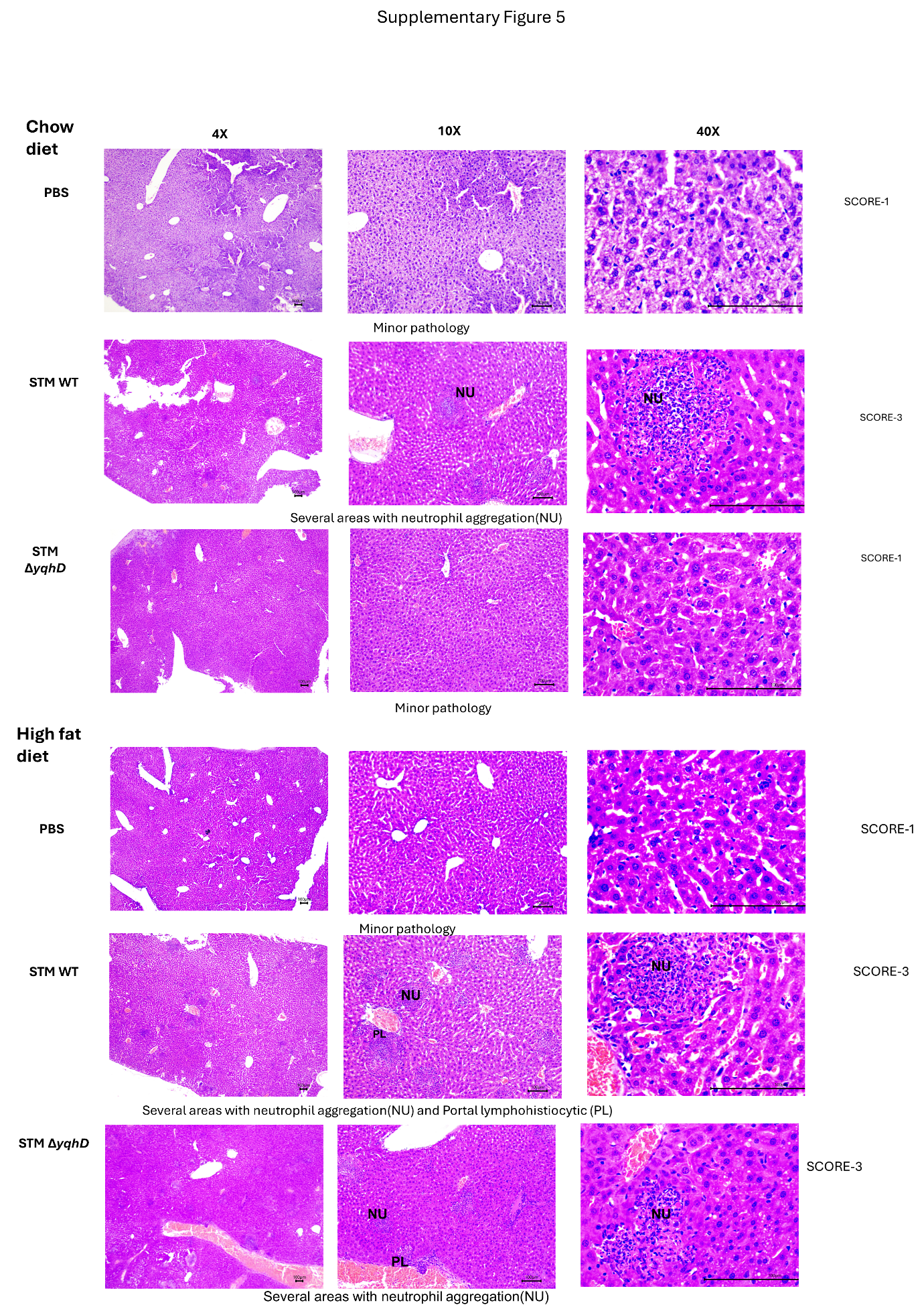


Histopathology images of liver sections used in the main figure 2 at different magnifications with their pathology scores. Scaler bars used in images is 100µm.

**Supplementary Fig.6. High-fat diet increases organ burden of STM Δ*yqhD* in C57BL/6 female mice**.


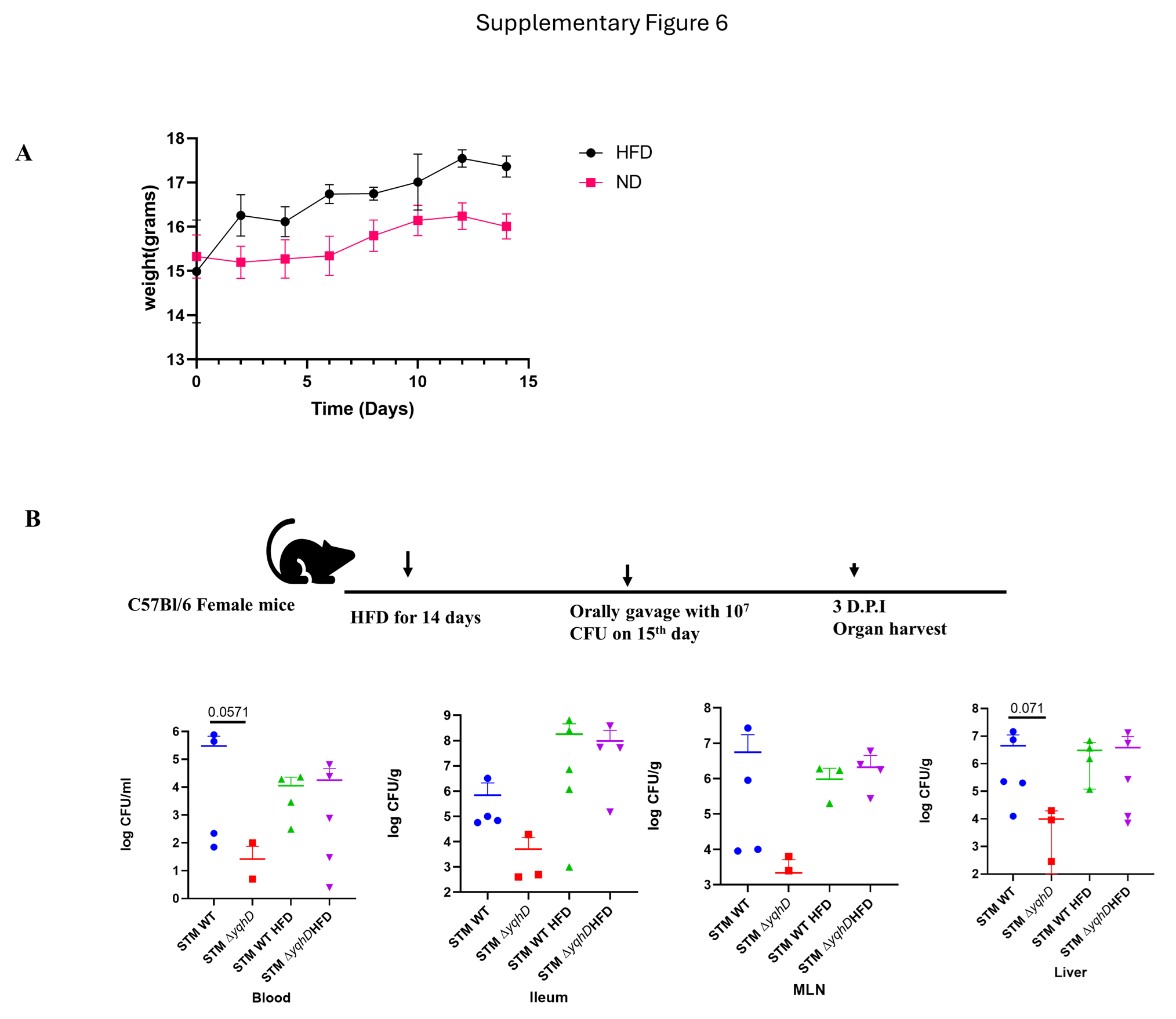


**A**. Average female mice weight on treatment with HFD compared to chow diet (ND) for 15 days.

**B**. Schematic of infection and organ burden of STM WT and STM Δ *yqhD* in C57BL/6 female mice 3 days post-infection, five animals were taken for each cohort, analysis performed by Mann- Whitney test, p values****<0.0001, ***<0.001, **<0.01, *<0.05.

**Supplementary Fig.7.** **Oleic acid treatment and *Salmonella* colonisation in C57BL/6 mice.**


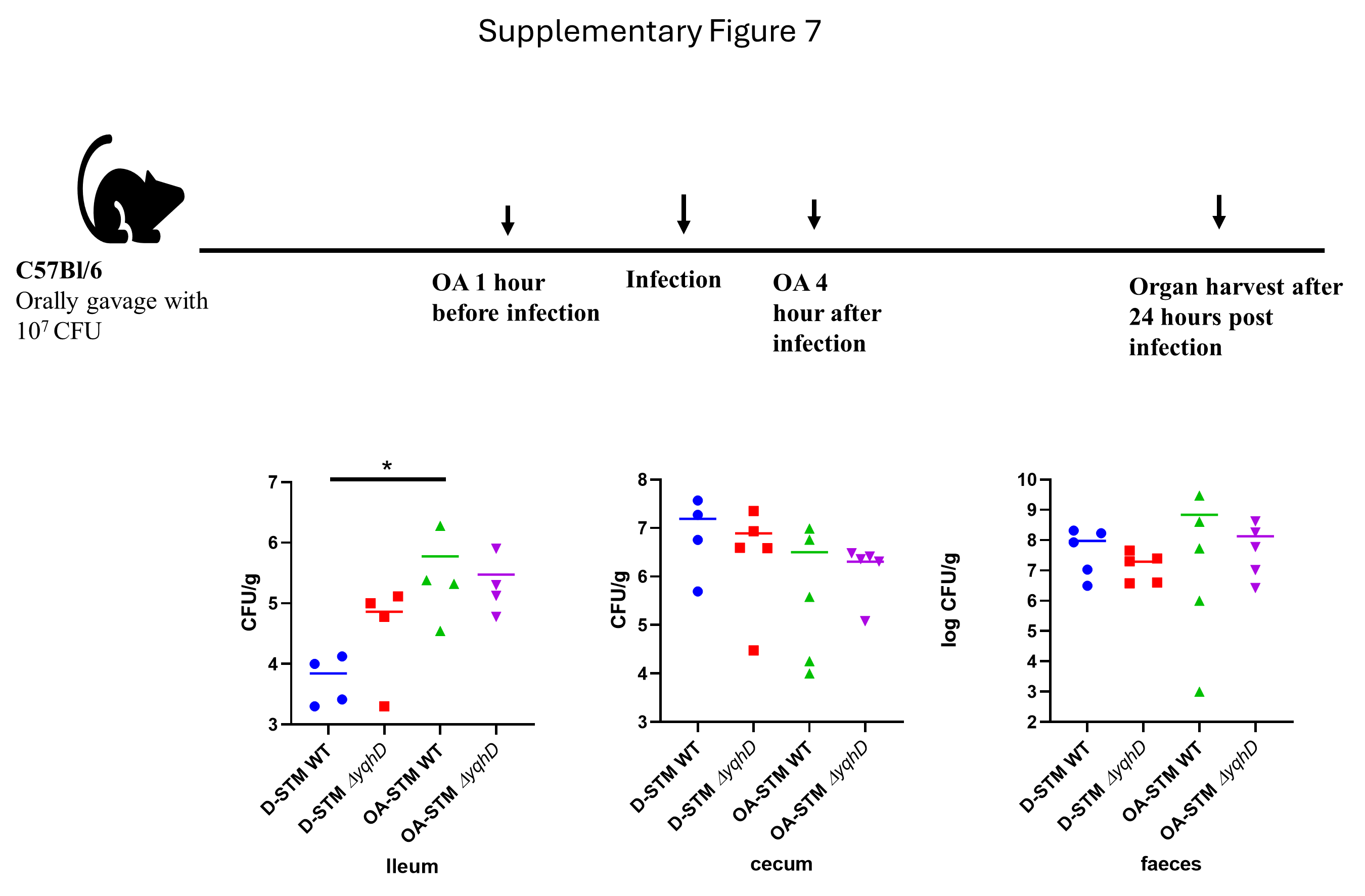


Schematic representation of animal infection and bacterial organ burden of STM WT, STMΔ*yqhD* in the ileum, Cecum and Faeces, five animals were taken for each cohort , analysis by Mann- Whitney test. p values****<0.0001, ***<0.001, **<0.01, *<0.05.

**Supplementary Fig .8. Haematoxylin and eosin staining of the liver sections from *gp91^phox-/-^*  mice at various magnifications with the pathology score.**


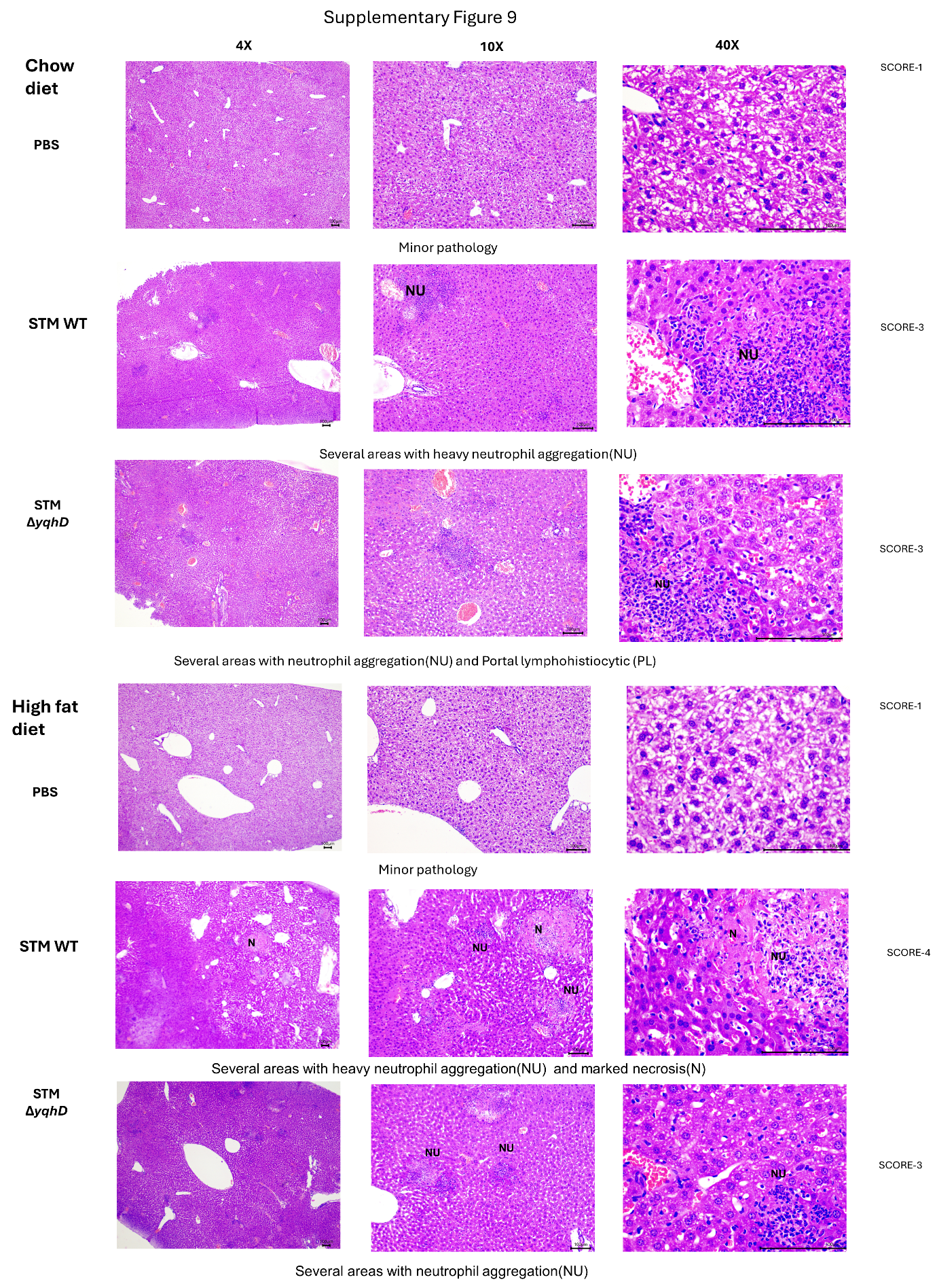


Histopathology images of liver sections used in the main figure 4 at different magnifications with their pathology scores. Scale bar used in the images is 100µm.

**Supplementary Fig 9. Spot assay on the plate with LB-agar or LB-agar supplemented with 7% bile.**


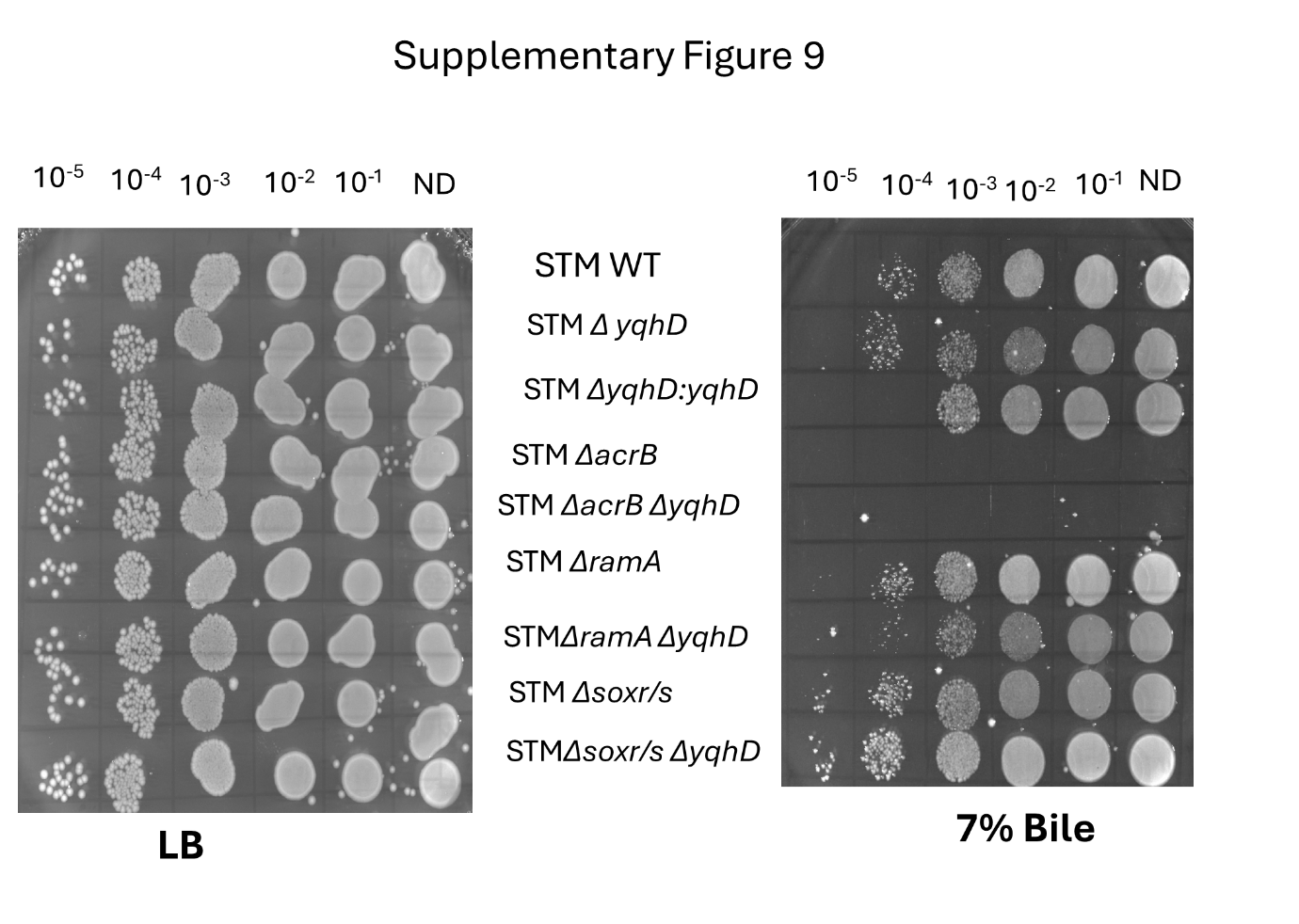


Spot assay of STM WT, STM *ΔyqhD ,*STM *ΔyqhD:yqhD,* STM *ΔacrB,* STM *ΔacrB ΔyqhD ,* STM *ΔramA ,*STM *ΔramA ΔyqhD,* STM *ΔsoxR/S and* STM *ΔsoxR/S ΔyqhD* (N=2).

Spot volume= 5 microlitres of 0.3 OD adjusted bacteria.

**Supplementary Fig.10 . Deletion of *yqhD* in *Salmonella* decreases invasion.**


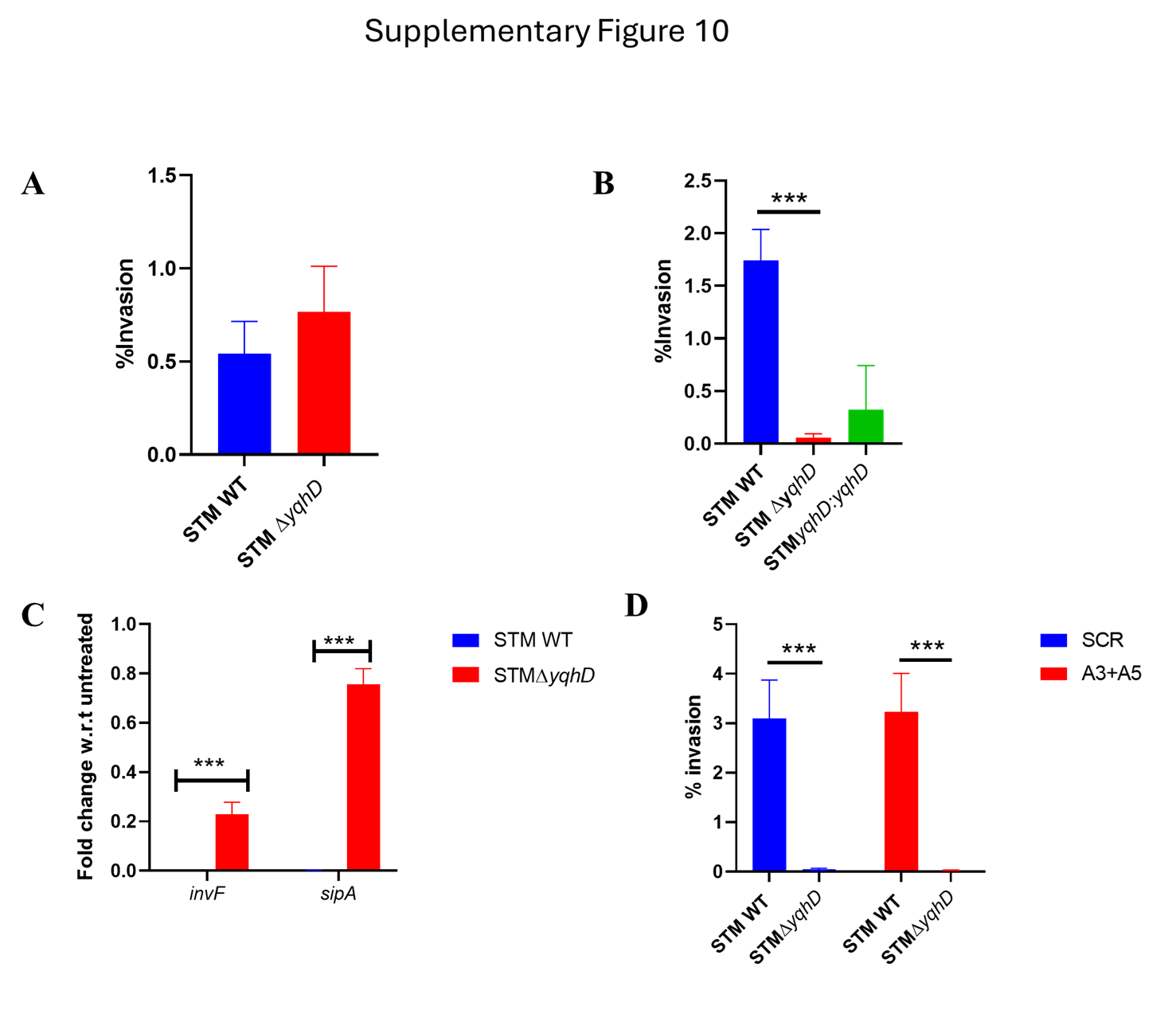


**A,B**. Invasion of STM WT, STM *ΔyqhD,* STM *ΔyqhD:yqhD* in colon carcinoma and HepG2 cells, respectively (N=4,n=3), data are presented with mean ± SD, analysis performed using unpaired Student's t-test. **C**.q-RT PCR of invasion genes on bile salt treatment in STM WT, STM *ΔyqhD* at 6 hours*.* Fold change has been calculated with respect to untreated samples grown in LB (N=2,n=3), data are presented with mean ± SD, analysis performed using two-way ANOVA..**D**. Percentage invasion in HepG2 cells with CYP7A1 knockdown (N=4,n=3), data are presented with mean ± SD. Analysis performed using two-way ANOVA.; p values****<0.0001, ***<0.001, **<0.01, *<0.05.

**Supplementary table S1. List of bacterial strains used in this study**

This table contains bacterial strains along with their respective antibiotics.

| **Table S1. List of the bacterial strains used in the study**:   \| S.NO \| Bacterial strain \| Antibiotic \| \| --- \| --- \| --- \| \| 1 \| *Salmonella* Typhimurium 14028S (STM WT) \| - \| \| 2 \| STM *ΔyqhD* \| kanamycin \| \| 3 \| STM *ΔyqhD:yqhD* \| Kanamycin, ampicillin \| \| 4 \| STM *ΔacrB* \| Chloramphenicol \| \| 5 \| STM *ΔacrB ΔyqhD* \| Kanamycin, Chloramphenicol \| \| 6 \| STM *ΔramA* \| Chloramphenicol \| \| 7 \| STM *ΔramA ΔyqhD* \| Kanamycin, Chloramphenicol \| \| 8 \| STM *ΔsoxR/S* \| Chloramphenicol \| \| 9 \| STM *ΔsoxR/S ΔyqhD* \| Kanamycin, Chloramphenicol \| \| 10 \| *Salmonella* Typhi CT 18(STY) \| - \| \| 11 \| STY *ΔyqhD* \| Kanamycin \| |
| --- | --- | --- | --- | --- | --- | --- | --- | --- | --- | --- | --- | --- | --- | --- | --- | --- | --- | --- | --- | --- | --- | --- | --- | --- | --- | --- | --- | --- | --- | --- | --- | --- | --- | --- | --- | --- |

**Supplementary table S2. List of primers used in this study**

This table contains the primer sequences for the genes used in the q-RT PCR (RT), knockout generation (KO) and complement strain generation.

**Table S2. List of primers used in the study**

| \| Primer name \| Primer sequence \| \| --- \| --- \| \| y*qhD* RT FP \| CTATCGTTCTGCCTGCGCTA \| \| *yqhD* RT RP \| TGCCATCCAGACCGTAATCG \| \| 16S RT FP \| CGCTTCTCTTTGTATGCGCC \| \| 16S RT RP \| TCGTCAGCTCGTGTTGTGAA \| \| *ompC* RT FP \| GGCAAAACCGTAAGAGGTGGA \| \| *ompC* RT RP \| AACGTACTGCCGATCAGAAC \| \| *ompF* RT FP \| CTGGTACTGAGCGACCACTTC \| \| *ompF* RT RP \| ATGGCGATCGCGCAGAATCGT \| \| *rfaG* RT FP \| GGAATGGTTCAGTCATCGCTT \| \| *rfaG* RT RP \| CGTAATGATATCGCGGAGTTA \| \| *ybis* RT FP \| ACAGCGGGTTATGCACCTCA \| \| *ybis* RT RP \| ATGCTATTCACGGCACCAACG \| \| *wecD* RT FP \| AAGGGCATCGCGACCAGCAT \| \| *wecD* RT RP \| TTCCCGCAGCGAGACGTAAC \| \| *acrR* RT FP \| AGTCGGATTTATTCAGTGAG \| \| *acrR* RT RP \| GCCTGTTGAACCACAACCATT \| \| *dinP* RT FP \| AACCGCTCTCGCTGGATGAA \| \| *dinP* RT RP \| AACATATTGTCCGTTCGGT \| \| *mcrB* RT FP \| TACGGCGTCTATCTGGATCA \| \| *mcrB* RT RP \| ATGCTATTGGCCTGCACGGT \| \| *recA* RT FP \| TTGATGCCTTCACCGTAGAG \| \| *recA* RT RP \| TTGGCGTGATGTTCGGTAACC \| \| *ram A* RT FP \| CAGGTTATCGACACGATTGT \| \| *ramA* RT RP \| TCCGTTCACGCACGTAGCGT \| \| *soxR* RT FP \| GTTGCGAAACGTAGCGGTGTT \| \| *soxR* RT RP \| TACCAAACGCGTCGCCGATA \| \| *yqhD* KO FP \| CAGCATTCTGGAGAAATAGGCAAAACATTGGCAGAAATGTGCATATGAATATCCTCCTTAG \| \| *yqhD* KO RP \| AAACCTTAGCGCGCCGCTTCATAAATCCGACGGCTGACAGTGTAGGCTGGAGCTGCTTC \| \| *yqhD* conf. FP \| TCCGCTGGATTGTGCGCATT \| \| *yqhD* conf. RP \| GATAATGGTTGGATTAGCCA \| \| *acrB* KO FP \| ACCAGCAGTGACGATCTCCGCAACCTACCCTGGCGCTGAT CATATGAATATCCTCCTTAG \| \| *acrB* KO RP \| ATTAAGTCTTTGGCGAATTCGACGATAAGTATCGCGTTCT GTGTAGGCTGGAGCTGCTTC \| \| *acrB* conf. FP \| TCCTCAAATTGCCGGTAGCG \| \| *acrB* conf. RP \| CTCCAGCGTCGCTTCTACCA \| \| *ramA* KO FP \| ATGACCATTTCCGCTCAGGTTATCGACACGATTGTCGAGTCATATGAATATCCTCCTTAG \| \| *ramA* KO RP \| TTTTCTTTACGATAAGCGCCTGGCGGCAGGTTGAACGTGGTGTAGGCTGGAGCTGCTTC \| \| *ramA* conf. FP \| GCTGACGAGTTTGATAGAGG \| \| *ramA* conf. RP \| TTCGCTGGCCGATTAAACATT \| \| *soxR/S* KO FP \| GGCGGAATACACGCGAGAAGGTTTGCTGCGATACATAGCC CATATGAATATCCTCCTTAG \| \| *soxR/S* KO RP \| CGCCTGGATTTCGCAGCGGACAGTCGCTACGCGATAAACAGTGTAGGCTGGAGCTGCTTC \| \| *soxR/s* conf. FP \| TTCATCGCCTGGCTACAACAA \| \| *soxR/s* conf. RP \| ACAAAGACCGGAAACAAACT \| \| *yqhD* complement FP \| CCGGAATTCCCCACATAAGGGAATGAGCA \| \| *yqhD* complement RP \| CCCAAGCTTAAACGAAAGTCAGAGGCGAA \| |  |  |  |  |  |  |
| --- | --- | --- | --- | --- | --- | --- | --- | --- | --- | --- | --- | --- | --- | --- | --- | --- | --- | --- | --- | --- | --- | --- | --- | --- | --- | --- | --- | --- | --- | --- | --- | --- | --- | --- | --- | --- | --- | --- | --- | --- | --- | --- | --- | --- | --- | --- | --- | --- | --- | --- | --- | --- | --- | --- | --- | --- | --- | --- | --- | --- | --- | --- | --- | --- | --- | --- | --- | --- | --- | --- | --- | --- | --- | --- | --- | --- | --- | --- | --- | --- | --- | --- | --- | --- | --- | --- | --- | --- | --- | --- | --- | --- | --- | --- | --- | --- |
| \| 18S human RT FP \| TTTCGGAACTGAGGCCATGA \| \| --- \| --- \| \| 18S human RT RP \| GAACCTCCGACTTTCGTTCTTGA \| \| Cyp7A1 RT FP \| TGGTGCCAATCCTCTTGAGT \| \| Cyp7A1 RT RP \| TCTGTGCCCAAATGCCTTC \| \| *acrB* RT FP \| CTCTATCACCATCGTATCGG \| \| *acrB* RT RP \| AGTAATCCGTGACCTCATCC \| |  |  |  |  |  |  |

| **Supplementary table S3. Composition of High-fat diet**  This table contains the nutritional information on components of the high-fat diet used in the animal experiments.  **Table S3. Composition of High fat diet**-   \|  \|  \|  \| \| --- \| --- \| --- \| \| Nutrient information \|  \| High-fat diet (% kcal from) \| \|  \|  \|  \| \| Protein \|  \| 20 \| \| Carbohydrate \|  \| 20 \| \| Lipid \|  \| 60 \| \|  \|  \|  \| \| Formula \|  \| g/Kg \| \| Casein \|  \| 200 \| \| L-Cystine \|  \| 3 \| \| Corn starch \|  \| 0 \| \| Maltodextrine \|  \| 125 \| \| Sucrose \|  \| 68.8 \| \| Cellulose \|  \| 50 \| \| Soybean oil \|  \| 25 \| \| Lard \|  \| 245 \| \| Mineral mix \|  \| 35 \| \| Vitamin mix \|  \| 10 \| \| Choline bitartrate \|  \| 2 \| \| TBHQ \|  \| 0.008 \| \|  \|  \|  \| |  |  |  |  |  |  |
| --- | --- | --- | --- | --- | --- | --- | --- | --- | --- | --- | --- | --- | --- | --- | --- | --- | --- | --- | --- | --- | --- | --- | --- | --- | --- | --- | --- | --- | --- | --- | --- | --- | --- | --- | --- | --- | --- | --- | --- | --- | --- | --- | --- | --- | --- | --- | --- | --- | --- | --- | --- | --- | --- | --- | --- | --- | --- | --- | --- | --- | --- | --- | --- | --- | --- | --- | --- | --- | --- |

|  |  | | | | | |
| --- | --- | --- | --- | --- | --- | --- |
|  |  | | | | | |
|  |  |  |  |  |  |  |
|  |  |  |  |  |  |  |
|  |  |  |  |  |  |  |
|  |  |  |  |  |  |  |
|  |  | | | | | |
|  |  | | | | | |
|  |  |  |  |  |  |  |
|  |  |  |  |  |  |  |
|  |  |  |  |  |  |  |
